# A single bacterial genus maintains root growth in a complex microbiome

**DOI:** 10.1038/s41586-020-2778-7

**Published:** 2020-09-30

**Authors:** Omri M. Finkel, Isai Salas-González, Gabriel Castrillo, Jonathan M. Conway, Theresa F. Law, Paulo José Pereira Lima Teixeira, Ellie D. Wilson, Connor R. Fitzpatrick, Corbin D. Jones, Jeffery L. Dangl

**Affiliations:** 1 Department of Biology, University of North Carolina at Chapel Hill, Chapel Hill, NC, USA.; 2 Howard Hughes Medical Institute, University of North Carolina at Chapel Hill, Chapel Hill, NC, USA.; 3 Curriculum in Bioinformatics and Computational Biology, University of North Carolina at Chapel Hill, Chapel Hill, NC, USA.; 4 Department of Genetics, University of North Carolina at Chapel Hill, Chapel Hill, NC, USA.; 5 Lineberger Comprehensive Cancer Center, University of North Carolina at Chapel Hill, Chapel Hill, NC, USA.; 6 Carolina Center for Genome Sciences, University of North Carolina at Chapel Hill, Chapel Hill, NC, USA.; 7 Curriculum in Genetics and Molecular Biology, University of North Carolina at Chapel Hill, Chapel Hill, NC, USA.; 8 Department of Microbiology and Immunology, University of North Carolina at Chapel Hill, Chapel Hill, NC, USA.; 9 Present address: Department of Plant and Environmental Sciences, Institute of Life Science, The Hebrew University of Jerusalem, Jerusalem, Israel.; 10 Present address: Future Food Beacon of Excellence, School of Biosciences, University of Nottingham, Sutton Bonington, UK.; 11 Present address: Department of Biology, ‘Luiz de Queiroz’ College of Agriculture (ESALQ), University of São Paulo (USP), Piracicaba, Brazil.; 12 These authors contributed equally: Omri M. Finkel, Isai Salas-González, Gabriel Castrillo, Jonathan M. Conway.

## Abstract

Plants grow within a complex web of species that interact with each other and with the plant^1–10^. These interactions are governed by a wide repertoire of chemical signals, and the resulting chemical landscape of the rhizosphere can strongly affect root health and development^7–9,11–18^. Here, to understand how interactions between microorganisms influence root growth in *Arabidopsis*, we established a model system for interactions between plants, microorganisms and the environment. We inoculated seedlings with a 185-member bacterial synthetic community, manipulated the abiotic environment and measured bacterial colonization of the plant. This enabled us to classify the synthetic community into four modules of co-occurring strains. We deconstructed the synthetic community on the basis of these modules, and identified interactions between microorganisms that determine root phenotype. These interactions primarily involve a single bacterial genus (*Variovorax*), which completely reverses the severe inhibition of root growth that is induced by a wide diversity of bacterial strains as well as by the entire 185-member community. We demonstrate that *Variovorax* manipulates plant hormone levels to balance the effects of our ecologically realistic synthetic root community on root growth. We identify an auxin-degradation operon that is conserved in all available genomes of *Variovorax* and is necessary and sufficient for the reversion of root growth inhibition. Therefore, metabolic signal interference shapes bacteria–plant communication networks and is essential for maintaining the stereotypic developmental programme of the root. Optimizing the feedbacks that shape chemical interaction networks in the rhizosphere provides a promising ecological strategy for developing more resilient and productive crops.

Plant phenotypes, and ultimately fitness, are influenced by the microorganisms that live in close association with them^1–3^. These microorganisms—collectively termed the plant microbiota—assemble on the basis of plant and environmentally derived cues^1,4–6^, resulting in myriad interactions between plants and microorganisms. Beneficial and detrimental microbial effects on plants can be either direct^3,7–9^, or an indirect consequence of microorganism–microorganism interactions^2,10^. Although antagonistic interactions between microorganisms are known to have an important role in shaping plant microbiota and protecting plants from pathogens^2^, another potentially important class of interactions is metabolic signal interference^11,12^: rather than direct antagonism, microorganisms interfere with the delivery of chemical signals produced by other microorganisms, which alters plant-microorganism signalling^12–14^.

Plant hormones—in particular, auxins—are both produced and degraded by an abundance of plant-associated microorganisms^15–18^. Microbially derived auxins can have effects on plants that range from growth promotion to the induction of disease, depending on context and concentration^9^. The intrinsic root developmental patterns of the plant are dependent on finely calibrated auxin and ethylene concentration gradients with fine differences across tissues and cell types^19^; it is not known how the plant integrates exogenous, microbially derived auxin fluxes into its developmental plan.

Here we apply a synthetic community to axenic plants as a proxy for root-associated microbiomes in natural soils to investigate how interactions between microorganisms shape plant growth. We establish plant colonization patterns across 16 abiotic conditions to guide stepwise deconstruction of the synthetic community, which led to the identification of multiple levels of microorganism–microorganism interaction that interfere with the additivity of bacterial effects on root growth. We demonstrate that a single bacterial genus (*Variovorax*) is required for maintaining the intrinsically controlled developmental programme of the root, by tuning its chemical landscape. We establish *Variovorax* as a core taxon in the root microbiota of diverse plants grown in diverse soils. Finally, we identify a locus conserved across *Variovorax* strains that is responsible for this phenotype.

## Microbial interactions control root growth

**Extended Data Fig. 1 |:**
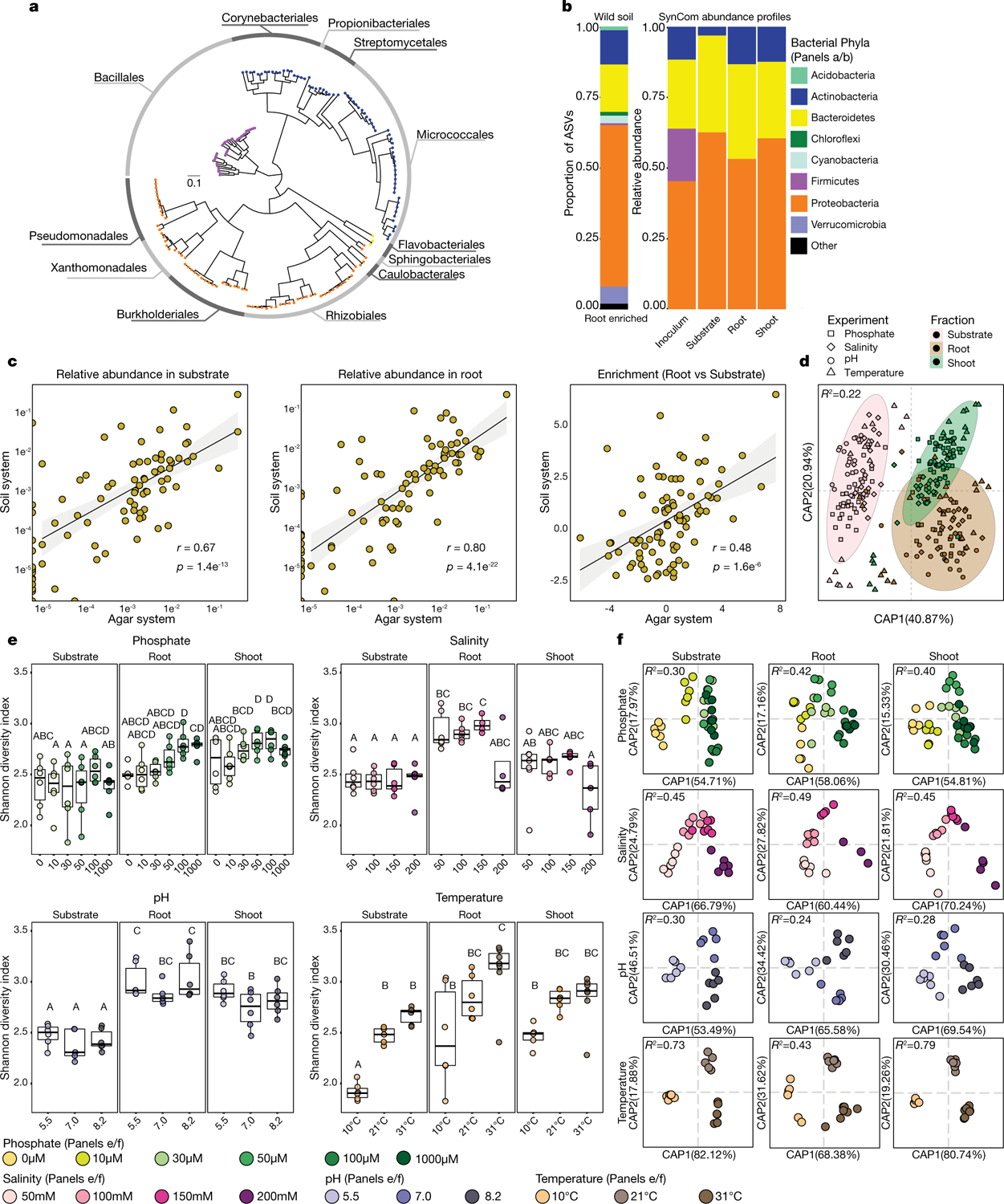
Synthetic community resembles the taxonomic make-up of natural communities. **a**, Phylogenetic tree of 185 genome-sequenced isolates obtained from surface-sterilized *Arabidopsis* roots, included in the synthetic community. The composition of this synthetic community captures the diversity of Actinobacteria, Proteobacteria and Bacteroidetes; the three major root-enriched phyla^1,4–6,20^; and Firmicutes, which are abundant in plant-associated culture collections^20^. Tree tips are coloured according to phylum. The outer ring shows the distribution of the 12 bacterial orders present in the synthetic community. **b**, Comparison of proportions of Proteobacteria, Bacteroidetes and Actinobacteria in synthetic-community (SynCom)-inoculated roots to root microbiota derived from plants grown in natural soil^1^. Firmicutes, which are not plant-enriched, were reduced to <0.1% of the relative abundance (Fig. 1a). The left panel (wild soil) shows the proportion of ASVs enriched (*q* value < 0.1) in the plant root in comparison to soil in a microbiota profiling study from the same soil from which the synthetic community strains were isolated. ASVs are coloured according to phylum and proteobacterial ASVs are coloured by class. The right panel (synthetic community) represents the relative abundance profiles of bacterial isolates across the initial inoculum, planted agar, and root and shoot in plants inoculated with the full synthetic community. **c**, Comparison of synthetic community composition in agar versus the soil-based microcosms. Left, relative abundance in the substrate. Middle, relative abundance in root. Right, enrichment in root versus substrate. Each dot represents a single unique sequence. Pearson correlation line, 95% confidence intervals, *r* value and *P* value are shown for each comparison. *n* = 24 (soil system) and 8 (agar system) biological replicates. **d**, Canonical analysis of principal coordinates (CAP) showing the influence of the fraction (planted agar, root or shoot) on the assembly of the bacterial synthetic community across the four gradients used in this Article (phosphate, salinity, pH and temperature). Different colours differentiate between the fractions, and different shapes differentiate between experiments. Ellipses denote the 95% confidence interval of each fraction. Fraction (substrate, root or shoot) explains most (22%) of the variance across all abiotic variables. *n* = 94 (substrate), 90 (root) and 95 (shoot) biological replicates across 8 independent experiments. **e**, Abiotic conditions displayed reproducible effects on α-diversity. Each panel represents bacterial α-diversity across the different abiotic gradients (phosphate, salinity, pH and temperature) and fractions (substrate, root and shoot) used in this Article. Bacterial α-diversity was estimated using Shannon diversity index. Letters represent the results of the post hoc test of an ANOVA model testing the interaction between fraction and abiotic condition. **f**, Canonical analysis of principal coordinates scatter plots showing the effect on the composition of the synthetic community of each of the four abiotic gradients (phosphate, salinity, pH and temperature) within the substrate, root and shoot fractions. PERMANOVA *R*^2^ values are shown within each plot. **e**, **f**, Phosphate, *n* = 6, 5, 6, 6, 6, 6, 6, 6, 6, 6, 6, 6, 6, 6, 6, 6, 5 and 6; salinity *n* = 6, 6, 6, 6, 6, 6, 6, 4, 5, 6, 4 and 5; pH, *n* = 6, 6, 6, 5, 5, 6, 6, 6 and 6; temperature *n* = 6, 6, 6, 6, 6, 6, 6, 6 and 6. All samples are biologically independent and represent two independent experiments.

To model plant–microbiota interactions in a fully controlled setting, we established a plant–microbiota microcosm that represents the native bacterial root-derived microbiota on agar plates. We inoculated 7-day-old seedlings with a defined 185-member bacterial synthetic community^5^ composed of the major root-associated phyla^1,4–6,20^ (Extended Data Fig. 1a, Supplementary Table 1). We exposed this microcosm to each of 16 abiotic contexts by manipulating one of four variables (salinity, temperature, a previously reported phosphate concentration gradient^5^ and pH). We measured the composition of the synthetic community in root, shoot and agar fractions 12 days after inoculation using 16S rRNA amplicon sequencing.

The composition of the resulting root and shoot microbiota recapitulated phylum-level plant-enrichment patterns seen in soil-planted *Arabidopsis*^1^ (Extended Data Fig. 1b). We validated the patterns observed in the agar-based system using seedlings grown in sterilized potting soil^21^ that were inoculated with the same synthetic community. Both relative abundance and plant-enrichment patterns at the unique sequence level were significantly correlated between the agar- and soil-based systems, which confirms the applicability of our relatively high-throughput agar-based system as a model for the assembly of plant microbiota (Extended Data Fig. 1c). Within the agar system, both fraction (substrate, root or shoot) and abiotic conditions significantly affected α- and β-diversity (Extended Data Fig. 1d–f).

**Fig. 1 |:**
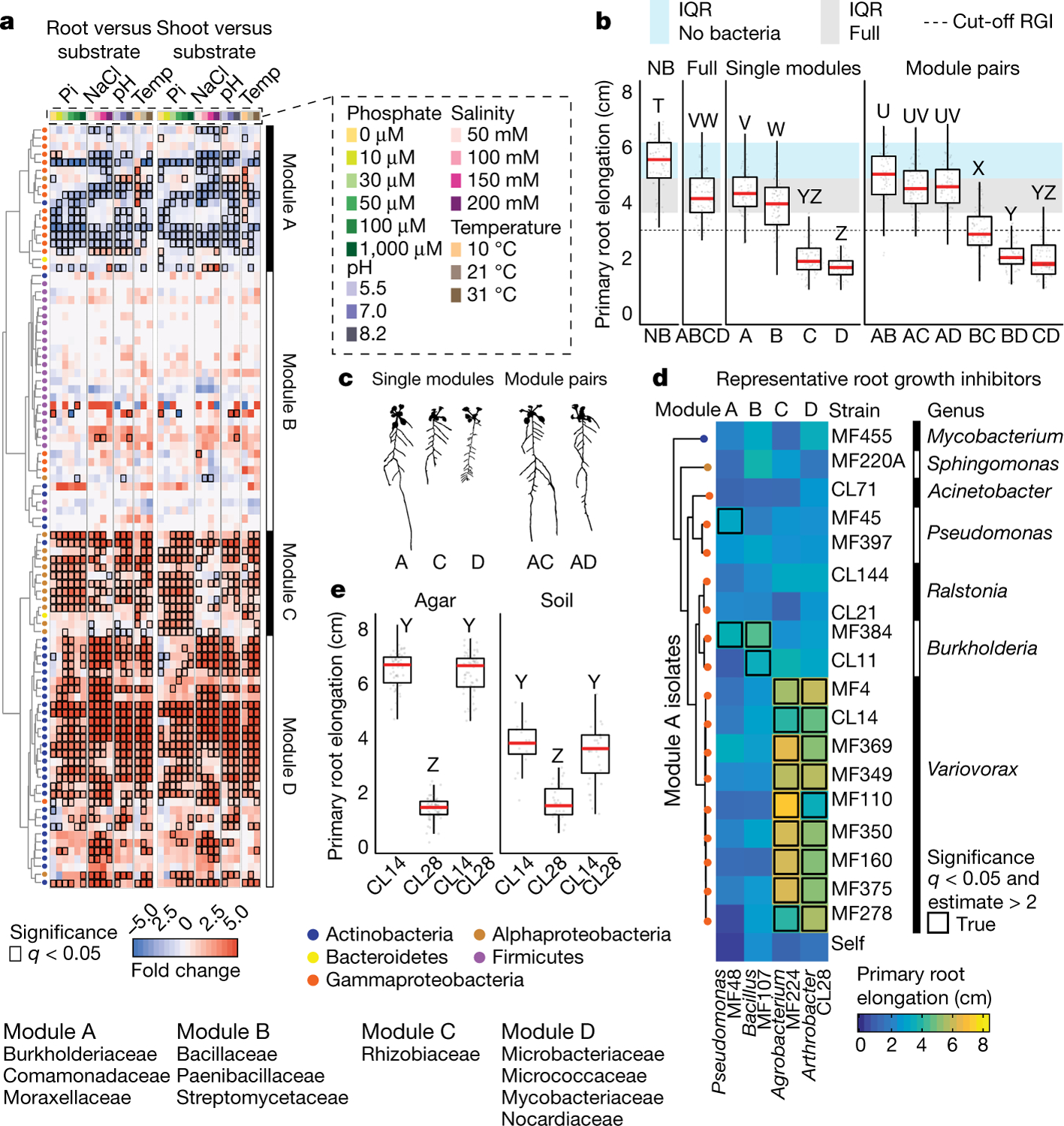
*Arabidopsis* root length is governed by bacteria–bacteria interactions within a community. **a**, Fraction enrichment patterns of the synthetic community across abiotic gradients. Each row represents a unique sequence. The four modules of co-occurring strains (A, B, C and D) are represented. Dendrogram tips are coloured by taxonomy. Heat maps are coloured by log_2_-transformed fold changes derived from a fitted generalized linear model and represent enrichments in plant tissue (root or shoot) compared with substrate. Comparisons with false-discovery-rate (FDR)-corrected *q*-value < 0.05 are contoured in black. Enriched families within each module are listed below the heat map. *n* = 6 biological replicates across 2 independent experiments. Pi, inorganic phosphate; temp, temperature. **b**, Primary root elongation of seedlings grown axenically (no bacteria, NB), with the full synthetic community (ABCD) or its subsets: modules A, B, C and D alone (single modules), and all pairwise combination of modules (module pairs). Significance was determined via analysis of variance (ANOVA); letters correspond to a Tukey post hoc test. *n* = 75, 89, 68, 94, 87, 77, 76, 96, 82, 84, 89 and 77 (from left to right) biological replicates across 2 independent experiments. **c**, Binarized image of representative seedlings inoculated with modules A, C and D, and with module combinations A–C and A–D. **d**, Heat map coloured by average primary root elongation of seedlings inoculated with four representative RGI-inducing strains from each module (columns A–D) in combination with isolates from module A (rows) or alone (self). Significance was determined via ANOVA. **e**, Primary root elongation of seedlings inoculated with *Arthrobacter* CL28 and *Variovorax* CL14 individually or jointly across two substrates. Significance was determined via ANOVA, letters are the results of a Tukey post hoc test. *n* = 64, 64, 63, 17, 36 and 33 (from left to right) biological replicates across 2 independent experiments. In all box plots, the centre line represents the median, box edges show the 25th and 75th percentiles, and whiskers extend to 1.5× the interquartile range (IQR).

To guide the deconstruction of the synthetic community into modules, we calculated pairwise correlations in relative abundance across all samples, and identified four well-defined modules of co-occurring strains that we termed modules A, B, C and D (Fig. 1a, Supplementary Table 2). These modules formed distinct phylogenetically structured guilds in association with the plant. Module A contained mainly Gammaproteobacteria and was predominantly more abundant in the substrate than in the seedling; module B contained mainly low-abundance Firmicutes, with no significant enrichment trend; and modules C and D were composed mainly of Alphaproteobacteria and Actinobacteria, respectively, and showed plant enrichment across all abiotic conditions. Both Alphaproteobacteria (module C) and Actinobacteria (module D) are consistently plant enriched across plant species^4^, which suggests that these clades contain plant-association traits that are deeply rooted in their evolutionary histories.

**Extended Data Fig. 2 |:**
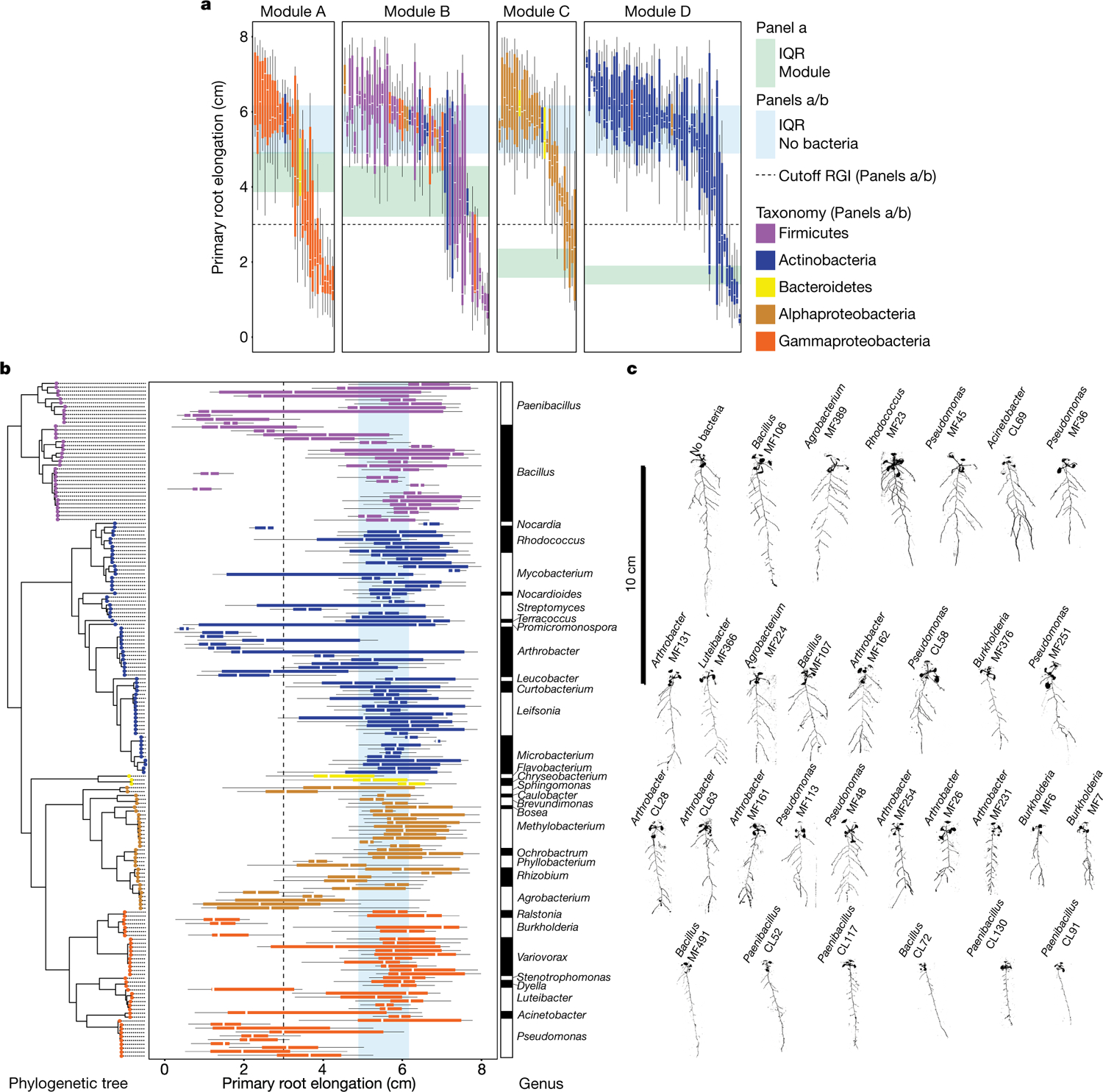
RGI trait is distributed across bacterial phylogeny. **a**, **b**, Primary root elongation of seedlings inoculated with single bacterial isolates (one box plot per isolate). Isolates are coloured by taxonomy. **a**, Isolates are grouped by module membership. The strips across the panels correspond to the interquartile range (IQR), as noted at the far right. The dotted line represents the cut-off used to classify isolates as root-growth inhibiting (cutoff RGI). **b**, Isolates are ordered according to the phylogenetic tree on the left, and coloured on the basis of their genome-based taxonomy. The vertical blue stripes across the panel correspond to the IQR of plants grown in sterile conditions. The vertical dotted line represents the 3-cm cut-off used to classify strains as RGI strains. The bar on the right denotes the genus classification of each isolate. **c**, Binarized image of representative seedlings grown axenically (no bacteria) or with 34 RGI strains individually.

We next asked whether the different modules of co-occurring strains have different roles in determining plant phenotype. We inoculated seedlings with synthetic communities composed of modules A, B, C and D singly or in all six possible pairwise combinations, and imaged the seedlings 12 days after inoculation. We observed strong primary root growth inhibition (RGI) in seedlings inoculated with plant-enriched module C or D (Fig. 1b, c). RGI did not occur in seedlings inoculated with module A or B, which do not contain plant-enriched strains (Fig. 1b). To test whether the root phenotype derived from each module is an additive outcome of its individual constituents, we inoculated seedlings in mono-association with each of the 185 members of the synthetic community. We observed that 34 taxonomically diverse strains, distributed across all 4 modules, induced RGI (Extended Data Fig. 2a–c). However, neither the full synthetic community nor derived synthetic communities that consisted of module A or B exhibited RGI (Fig. 1b). Thus, binary plant–microorganism interactions were not predictive of interactions in this complex-community context.

In seedlings inoculated with module pairs, we observed epistatic interactions: in the presence of module A, the RGI caused by modules C and D was reverted (Fig. 1b, c). Thus, by deconstructing the synthetic community into four modules, we found that bacterial effects on root growth are governed by multiple levels of microorganism–microorganism interaction. This is exemplified by at least four instances: within module A or B and between module A and module C or D. Because three of these interactions involve module A, we predicted that this module contains strains that strongly attenuate RGI and preserve stereotypic root development.

## *Variovorax* maintain stereotypic root growth

**Extended Data Fig. 3 |:**
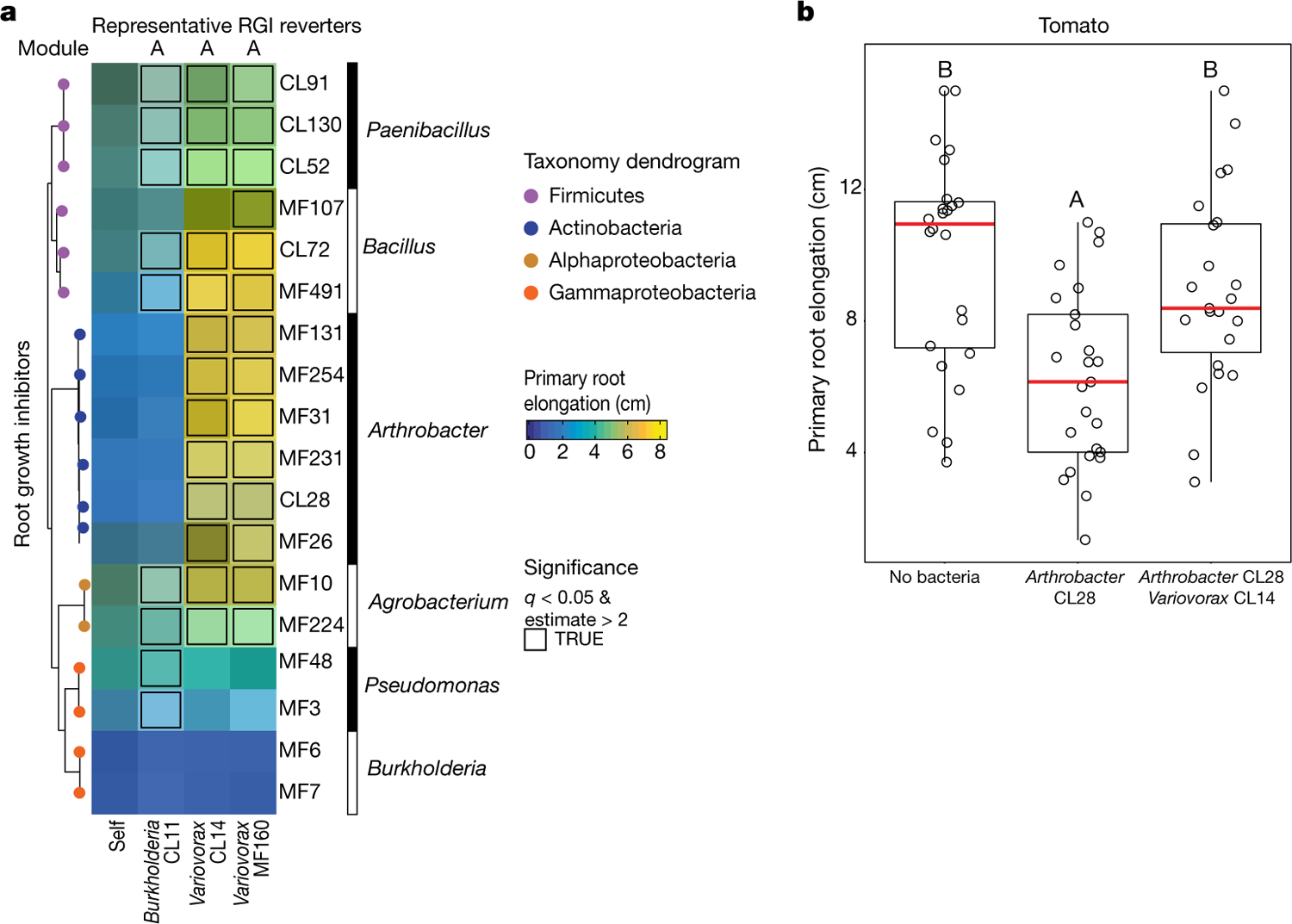
*Variovorax*-mediated reversion of RGI. **a**, Heat map coloured by average primary root elongation of seedlings inoculated with eighteen RGI-inducing strains (rows) alone (self) or in combination with *Burkholderia* CL11, *Variovorax* CL14 or *Variovorax* MF160 (columns). Statistically significant RGI reversions were determined via ANOVA and are outlined in black. **b**, *Variovorax*-mediated reversion of RGI is maintained in a second plant species. Primary root elongation of uninoculated tomato seedlings (no bacteria) or seedlings inoculated with the RGI-inducer *Arthrobacter* CL28 individually or along with *Variovorax* CL14 grown on vertical agar plates. Significance was determined via ANOVA while controlling for experiment; letters correspond to a Tukey post hoc test. *n* = 24, 25 and 23 biological replicates across 2 independent experiments.

To identify strains within module A that are responsible for intra- and intermodule attenuation of RGI, we reduced our system to a tripartite plant–microorganism–microorganism system. We individually screened the 18 non-RGI strains from module A for their ability to attenuate RGI caused by representative strains from all 4 modules. We found that all the tested strains from the genus *Variovorax* (family Comamonadaceae) suppressed the RGI caused by representative RGI-inducing strains from module C (*Agrobacterium* MF224) and module D (*Arthrobacter* CL28) (Fig. 1d). The strains from modules A (*Pseudomonas* MF48) and B (*Bacillus* MF107) were not suppressed by *Variovorax*, but rather by two closely related *Burkholderia* strains (CL11 and MF384) (Fig. 1d). A similar pattern was observed when we screened two selected RGI-suppressing *Variovorax* strains (CL14 and MF160) and *Burkholderia* CL11 against a diverse set of RGI-inducing strains. *Variovorax* attenuated 13 of the 18 RGI-inducing strains that we tested (Extended Data Fig. 3a).

**Extended Data Fig. 4 |:**
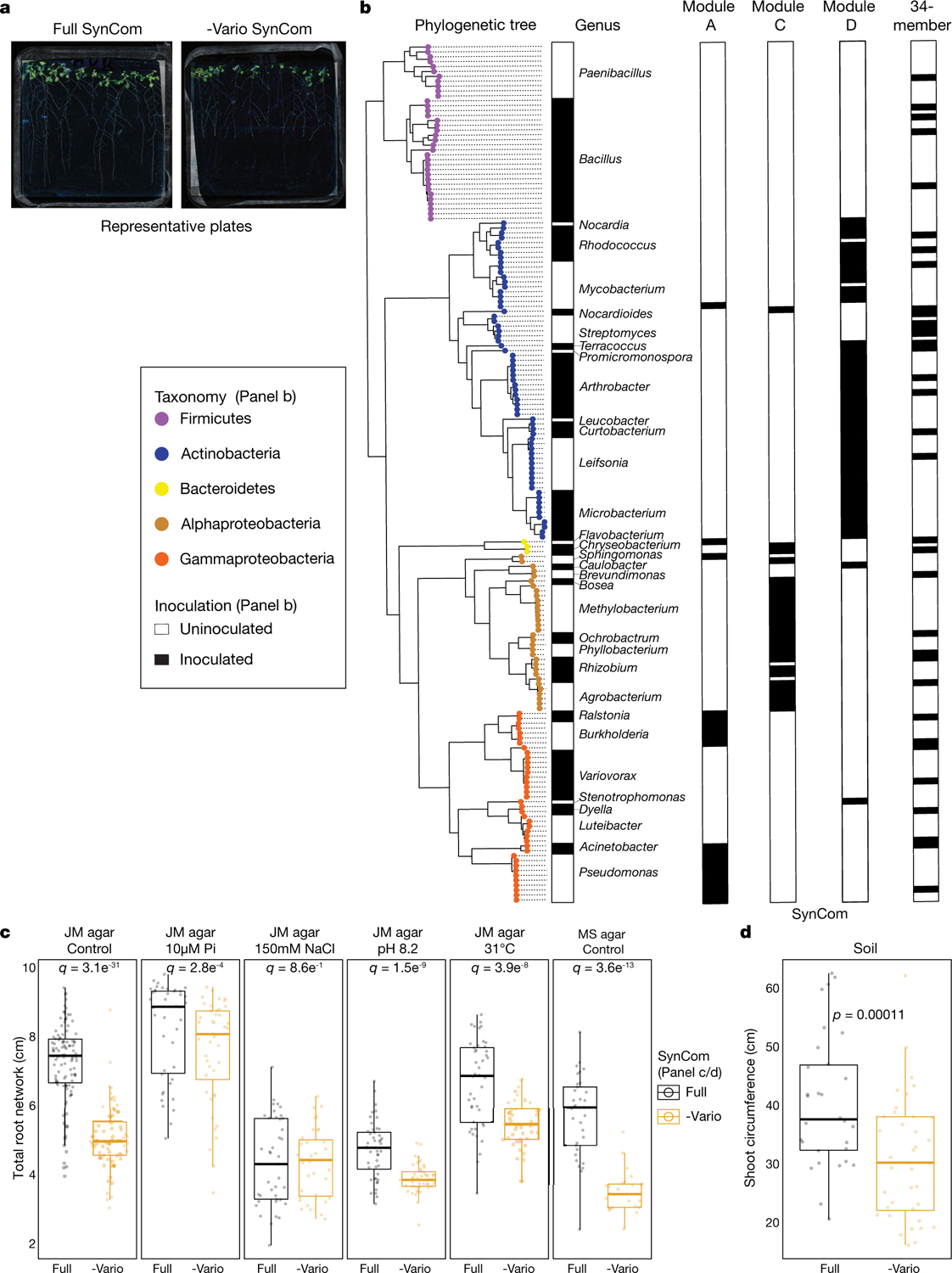
*Variovorax* maintain stereotypic plant growth. **a**, Representative plate images of plants grown with the full 185-member synthetic community (full SynCom) or the *Variovorax* drop-out community (−Vario SynCom) for 12 d. **b**, Bar graphs showing the isolate composition of synthetic communities composed by module A, module C, module D and a previously described^1^ 34-member synthetic community (34-member). Isolates are ordered according to the phylogenetic tree on the left. The tips of the phylogenetic tree are coloured on the basis of the genome-based taxonomy of each isolate. Presence of an isolate across the different synthetic communities is denoted by a black filled rectangle (labelled ‘inoculated’). **c**, Total root network quantification of *Arabidopsis* seedlings grown with the full synthetic community (full), or with the full synthetic community excluding *Variovorax* (−Vario), across different abiotic conditions: JM agar control, low phosphate (JM agar 10 μM Pi), high salt (JM agar 150 mM NaCl), high pH (JM agar pH 8.2) and high temperature (JM agar 31 °C), as well as half Murashige and Skoog medium (MS agar control). Significance was determined within each condition via ANOVA while controlling for experiment. *n* = 110, 77, 42, 44, 41, 35, 48, 49, 44, 45, 33 and 26 biological replicates across 2 independent experiments. **d**, Shoot circumference of plants grown in pots with potting soil with the full synthetic community or with the full synthetic community excluding *Variovorax*. Significance was determined within each condition via ANOVA while controlling for experiment. *n* = 30 and 36 biological replicates across 2 independent experiments.

**Fig. 2 |:**
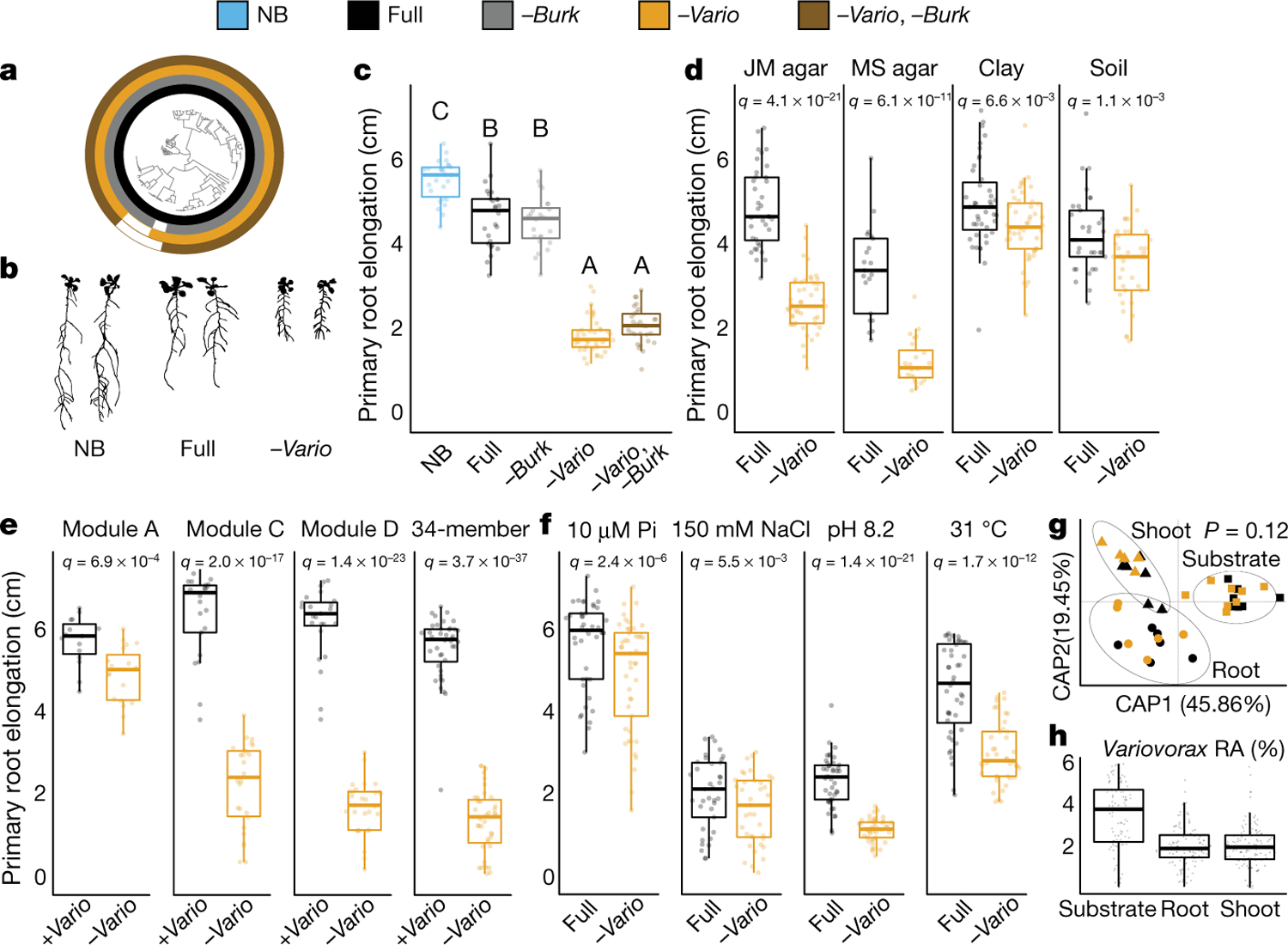
*Variovorax* maintains stereotypic root development. **a**, Phylogenetic tree of 185 bacterial isolates. Concentric rings represent isolate composition of each synthetic community treatment. NB, uninoculated (no bacteria); full, full synthetic community; −*Burk*, *Burkholderia* drop-out; −*Vario*, *Variovorax* drop-out; −*Vario*, −*Burk*, *Burkholderia* and *Variovorax* drop-out. **b**, Binarized image of representative seedlings inoculated or not with the full synthetic community or the *Variovorax* drop-out synthetic community. **c**, Primary root elongation of seedlings grown axenically (NB) or inoculated with the different synthetic community treatments outlined in **a**. Significance was determined via ANOVA, letters correspond to a Tukey post hoc test. *n* = 26, 26, 24, 37 and 29 (from left to right) biological replicates. **d**, Primary root elongation of seedlings inoculated with the full synthetic community or with the *Variovorax* drop-out synthetic community across different substrates: Johnson medium (JM) agar, Murashige and Skoog (MS) agar, sterilized clay or potting soil. *n* = 36, 47, 21, 24, 43, 48, 33 and 36 (from left to right) biological replicates across 2 independent experiments. **e**, Primary root elongation of seedlings inoculated with four subsets of the full synthetic community (module A, C, D or a previously described 35-member synthetic community with its single *Variovorax* strain removed (34-member)^1^), with (+*Vario*) or without (−*Vario*) *Variovorax* isolates. *n* = 40, 40, 15, 19, 22, 26, 25 and 29 (from left to right) biological replicates across 2 independent experiments. **f**, Primary root elongation of seedlings inoculated with the full synthetic community or with the *Variovorax* drop-out synthetic community across different abiotic stresses: low phosphate, high salt, high pH and high temperature. *n* = 43, 45, 37, 37, 43, 44, 44 and 43 (from left to right) biological replicates across 2 independent experiments. Significance was determined within each condition via ANOVA in **d**, **f**, and with a two-sided *t*-test in **e**. FDR-adjusted *P* values are displayed. **g**, Canonical analysis of principal coordinates (CAP) scatter plots comparing the compositions of the full synthetic community and *Variovorax* drop-out synthetic community (colour code as in **a**) across fractions (substrate (squares), root (circles) and shoot (triangles)). Permutational multivariate ANOVA (PERMANOVA) *P* value is shown. *n* = 7 (substrate + full), 8 (substrate + −*Vario*), 6 (root + full), 6 (root + −*Vario*), 5 (shoot + full) or 5 (shoot + −*Vario*). **h**, Relative abundance (RA) of the *Variovorax* genus within the full synthetic community across the agar, root and shoot fractions. *n* = 127, 119 and 127 biological replicates for agar, root and shoot, respectively, across 16 independent experiments.

To test whether the RGI induction and suppression we observed on agar occur in soil as well, we germinated *Arabidopsis* on sterile soil inoculated with an RGI-suppressing and -inducing pair of strains: the RGI-inducing *Arthrobacter* CL28 and the RGI-suppressing *Variovorax* CL14. As expected, *Arthrobacter* CL28 induced RGI, which was reverted by *Variovorax* CL14 in soil (Fig. 1e). We generalized this observation by showing that *Variovorax*-mediated attenuation of RGI extended to tomato seedlings, in which *Variovorax* CL14 reverted *Arthrobacter* CL28-mediated RGI (Extended Data Fig. 3b). Finally, we tested whether the RGI-suppressing strains maintain their capacity to attenuate RGI in the context of the full 185-member community. We compared the root phenotype of seedlings exposed to either the full synthetic community or to the same community after dropping out all ten *Variovorax* strains and/or all six *Burkholderia* strains present in the synthetic community (Fig. 2a). We found that *Variovorax* is necessary and sufficient to revert RGI within the full community (Fig. 2b, c, Extended Data Fig. 4a). This result was robust across a range of substrates (including soil), and under various biotic and abiotic contexts (Fig. 2d–f, Extended Data Fig. 4b). Further, the presence of *Variovorax* in the synthetic community increases both the total length of root network of the plant and its shoot size (Extended Data Fig. 4c, d). Importantly, the latter is considered a reliable proxy for relative plant fitness^22,23^, which suggests that *Variovorax*-mediated suppression of RGI is adaptive.

**Extended Data Fig. 5 |:**
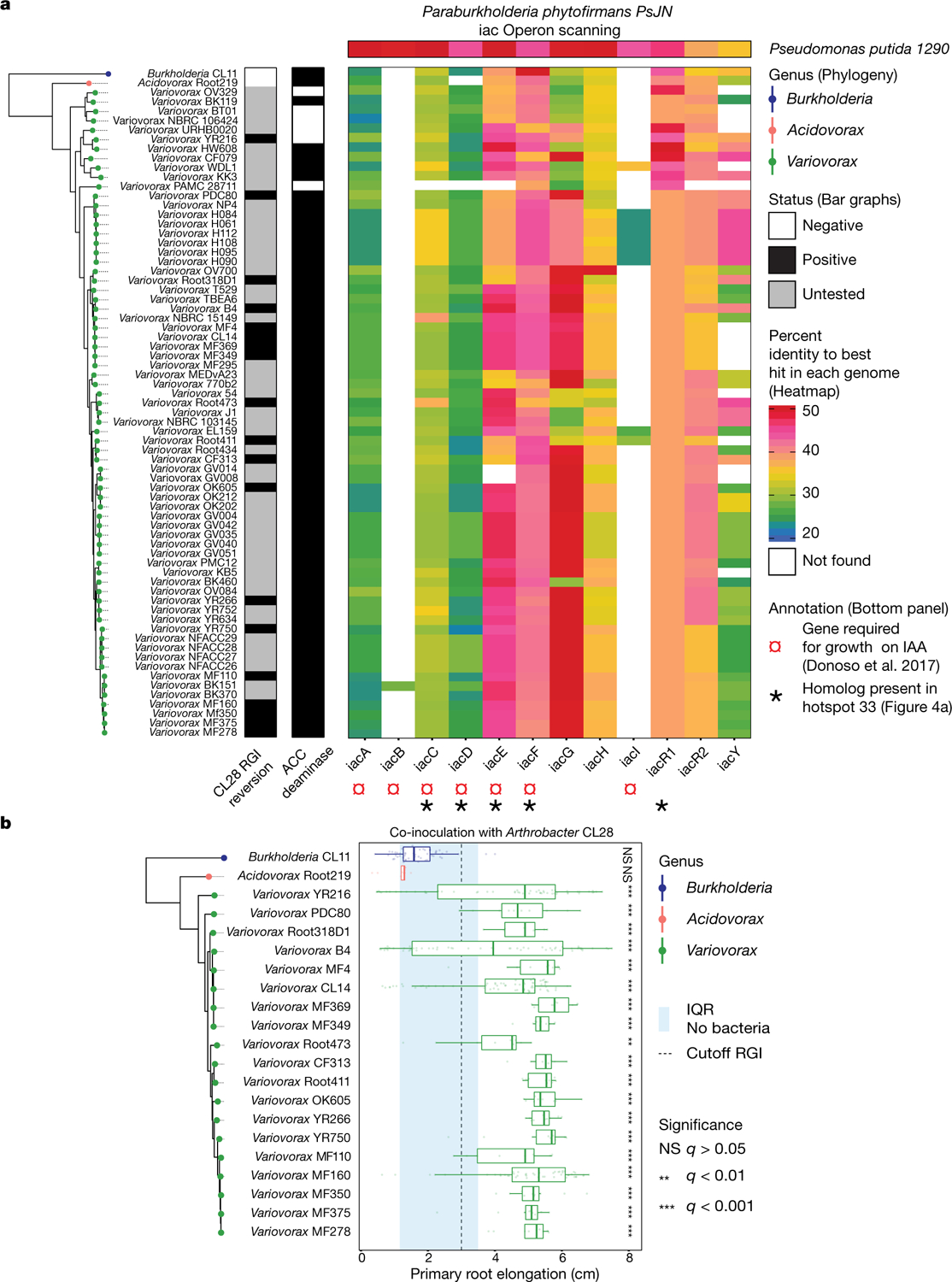
Reversion of RGI is prevalent across the *Variovorax* phylogeny. **a**, Phylogenetic tree of 69 publically available *Variovorax* genomes and 2 outgroup isolates, *Acidovorax* root 219 and *Burkholderia* CL11. The CL28 RGI reversion bar categorizes (positive, negative or untested) the ability of each isolate in the phylogeny to revert the RGI caused by *Arthrobacter* CL28. The ACC deaminase bar denotes the presence of the KEGG Orthology (KO) term KO1505 (1-aminocyclopropane-1-carboxylate deaminase) in each of the genomes. The heat map denotes the per cent identity of BLASTp hits in the genomes to the genes from the auxin-degrading *iac* operon in *Paraburkholderia phytophirmans*, described by ref.^17^. Synteny is not necessarily conserved, as these BLAST hits may be spread throughout the genomes. **b**,All tested *Variovorax* isolates reverted RGI. Phylogenetic tree of 19 *Variovorax* genomes and 2 outgroup isolates (*Acidovorax* root 219 and *Burkholderia* CL11) that were tested for their ability to revert the RGI imposed by *Arthrobacter* CL28. The blue vertical stripe denotes the IQR of plants treated solely with *Arthrobacter* CL28. The dotted vertical line denotes the 3-cm cut-off used to classify a treatment as an RGI. Each box plot is coloured according to the genus classification of each isolate. Significance was determined via ANOVA while controlling for experiment, letters correspond to a Tukey post hoc test. *n* = 59, 9, 55, 71, 10, 10, 10, 9, 10, 10, 57, 10, 10, 48, 10, 10, 10, 9, 10, 10 and 9 biological replicates across 2 independent experiments.

**Extended Data Fig. 6 |:**
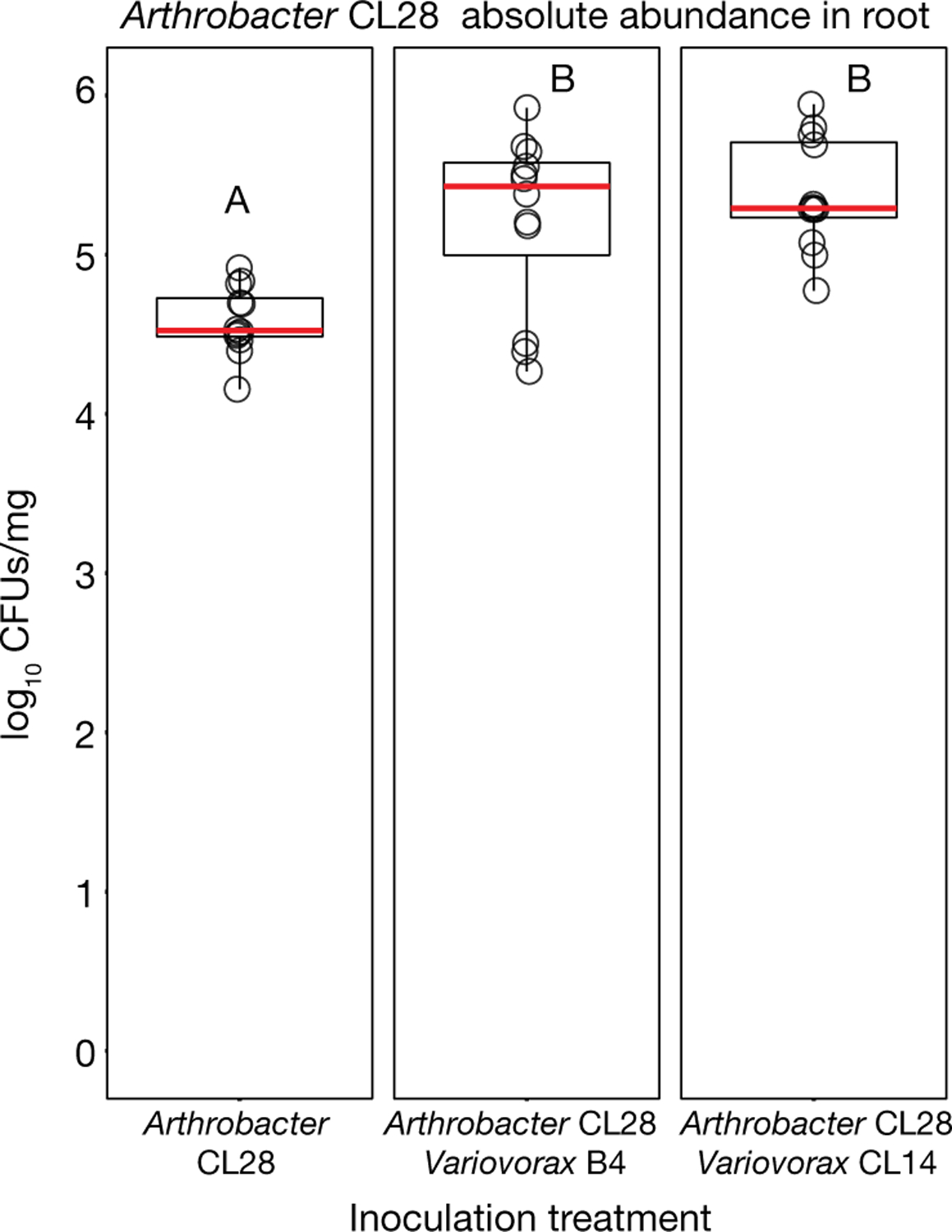
*Variovorax* does not compete with or antagonize RGI strains. To test whether *Variovorax* attenuates RGI by inhibiting the growth of RGI-inducing strains, we compared the bacterial relative abundance profiles in seedlings colonized with the full synthetic community to that of seedlings colonized with the *Variovorax* drop-out community. We found no changes in the abundances of RGI-inducing strains in response to the *Variovorax* drop-out (Fig. 2g). In addition, we measured in planta absolute abundance of *Arthrobacter* CL28 when inoculated alone or with two *Variovorax* representatives: *Variovorax* B4 and *Variovorax* CL14. Here we show log-transformed CFUs of *Arthrobacter* CL28 normalized to root weight. To selectively count *Arthrobacter* CL28, CFUs were counted on Luria Bertani (LB) agar plates containing 50 μg ml^−1^ of apramycin, on which neither *Variovorax* B4 nor *Variovorax* CL14 grow. *Arthrobacter* CL28 CFUs are not reduced in the presence of *Variovorax*. Notably, *Variovorax* account for only about 1.5% of the root community (Fig. 2h). These results rule out the possibility that *Variovorax* enforces stereotypic root growth by antagonizing or outcompeting RGI inducers. Significance was determined via ANOVA, letters correspond to a Tukey post hoc test. *n* = 12 biologically independent samples.

To ascertain the breadth of the ability of *Variovorax* to attenuate RGI, we tested additional *Variovorax* strains from across the phylogeny of this genus (Extended Data Fig. 5a, Supplementary Table 1). All 19 of the *Variovorax* strains we tested reverted the RGI induced by *Arthrobacter* CL28. A strain from the nearest plant-associated outgroup to this genus (*Acidovorax* root219^24^) did not revert RGI (Extended Data Fig. 5a, b). Thus, all tested strains—representing the broad phylogeny of *Variovorax*—interact with a wide diversity of bacteria to enforce stereotypic root development within complex communities, independent of biotic or abiotic contexts. Importantly, we found no evidence that this phenotype is achieved by outcompeting or antagonizing RGI-inducing strains (Fig. 2g, h, Extended Data Fig. 6).

## *Variovorax* manipulates auxin and ethylene

**Extended Data Fig. 7 |:**
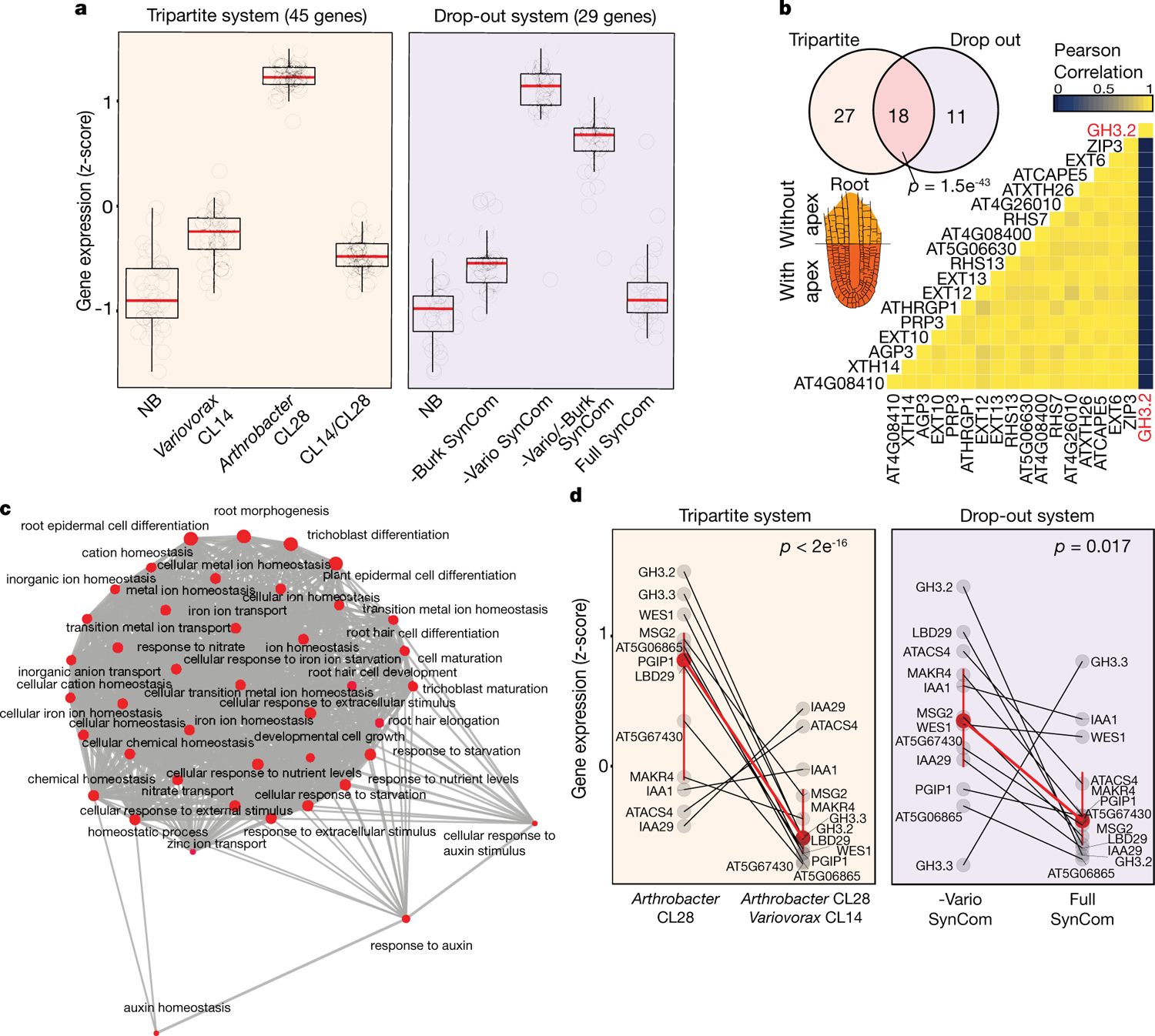
Auxin-responsive genes are induced in response to RGI strains. **a**, Box plots showing the average standardized expression of genes significantly induced only under RGI conditions (RGI-induced), across the following treatments. Left (tripartite system), uninoculated seedlings (NB) or seedlings inoculated with *Variovorax* CL14, *Arthrobacter* CL28 or both *Arthrobacter* CL28 and *Variovorax* CL14 (CL14/CL28). Right (drop-out system), uninoculated seedlings (NB), *Burkholderia* drop-out synthetic community (−Burk SynCom), *Variovorax* drop-out synthetic community (−Vario SynCom), *Variovorax* and *Burkholdria* drop-out synthetic community (−Vario −Burk SynCom) or the full synthetic community (full SynCom). RGI-induced genes are defined as genes that are significantly overexpressed in RGI treatments. Left, genes overexpressed in *Arthrobacter* CL28-inoculated seedling versus NB and in *Arthrobacter* CL28-inoculated seedlings versus CL14/CL28. Right, genes overexpressed in −Vario versus NB and in −Vario versus full. *n* = 3 (left); 5 (right). **b**, Venn diagram showing the overlap of enriched genes between the tripartite and drop-out systems. The heat map shows the pairwise correlation in expression of these 18 genes across tissues on the basis of the Klepikova Atlas^25^. Seventeen of the 18 genes show high correlation across the atlas, with the exception of the auxin-conjugating gene *GH3.2*. A root apex diagram from the *Arabidopsis* eFP Browser (http://bar.utoronto.ca/efp/cgi-bin/efpWeb.cgi)^68^ is shown, illustrating the spatial distribution of transcripts from the 17 highly correlated root apex-associated genes in the Klepikova Atlas^25^. Significance of the overlap of enriched genes was determined via hypergeometric test. **c**, RGI-related genes share gene ontologies. Network of statistically significant gene ontology terms contained within the 18 overlapping RGI-induced genes (**a**, **b**). The network was computed using the emapplot function from the package clusterProfiler in R. A *P* value for terms across the gene ontology was computed using a hypergeometric test (only significant ontologies are shown). Point size (gene ontology term) denotes the number of genes mapped to that particular term. **d**, Standardized expression of 12 late-responsive auxin genes^26^ across the tripartite and drop-out systems. Each dot represents a gene. Identical genes are connected between bacterial treatments with a black line. Mean expression (95% confidence intervals) of the aggregated 12 genes in each treatment is highlighted in red and connected between bacterial treatments with a red line. Significance was calculated using a resampling approach, in which we compared the calculated difference between means across groups. After 10,000 resamplings, we calculated the *P* value by comparing the distribution of means calculated against the real observed differences between means.

To study the mechanisms that underlie bacterial effects on root growth, we analysed the transcriptomes of seedlings colonized for 12 days with the RGI-inducing strain *Arthrobacter* CL28 and the RGI-suppressing strain *Variovorax* CL14, either in mono-association with the seedling or in a tripartite combination (Fig. 1e). We also performed RNA sequencing (RNA-seq) on seedlings colonized with the full synthetic community (no RGI) or the *Variovorax* drop-out synthetic community (RGI) (Extended Data Fig. 7a). Eighteen genes were significantly induced only under RGI conditions (RGI-induced) across both experiments (Extended Data Fig. 7a, b). Seventeen of these genes are co-expressed with genes that have proposed functions related to the root apex^25^ (Extended Data Fig. 7b, c). The remaining gene, *GH3.2*, encodes indole-3-acetic acid-amido synthetase, which conjugates excess amounts of the plant hormone auxin and is a robust marker for late auxin responses^26,27^ (Extended Data Fig. 7b). The production of auxins is a well-documented mechanism by which bacteria modulate plant root development^13^. Indeed, the top 12 auxin-responsive genes from a previous RNA-seq study examining acute auxin response in *Arabidopsis*^26^ exhibited an average transcript increase in seedlings exposed to our RGI-inducing conditions (Extended Data Fig. 7d). We hypothesized that the suppression of RGI by *Variovorax* is probably mediated by interference with bacterially produced auxin signalling.

**Extended Data Fig. 8 |:**
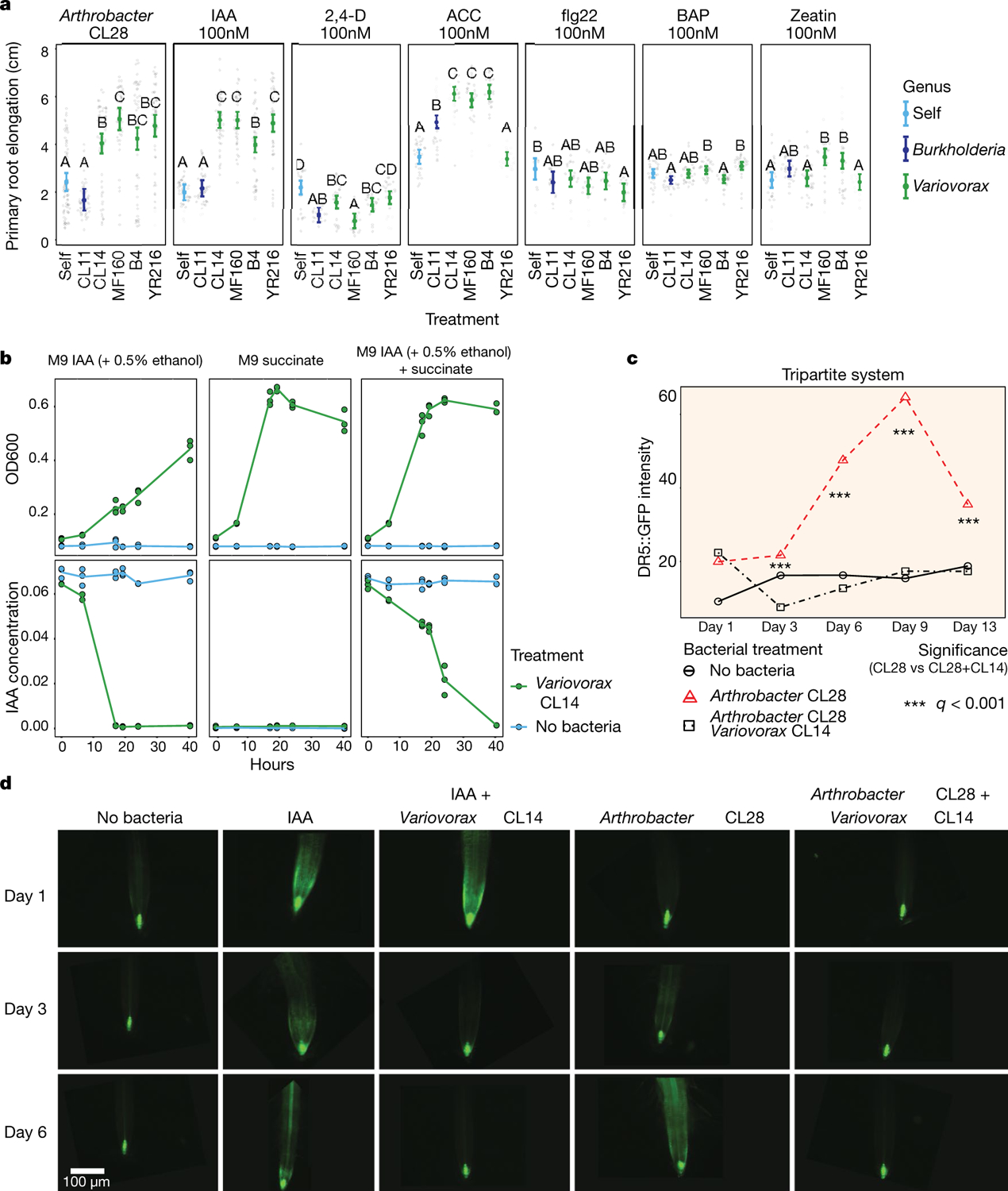
*Variovorax* degrades auxin and quenches auxin perception by the plant. **a**, Primary root elongation of seedlings grown with six hormonal or microbial-associated molecular pattern RGI treatments (panels) individually (self) or with either *Burkholderia* CL11 or each of four *Variovorax* isolates (CL14, MF160, B4 and YR216). Significance was determined via ANOVA within each panel; letters correspond to a Tukey post hoc test. *n* = 74, 46, 61, 48, 49, 49, 45, 44, 46, 43, 49, 40, 22, 19, 22, 19, 20, 25, 28, 30, 29, 29, 29, 29, 12, 12, 21, 20, 19, 18, 26, 30, 30, 29, 30, 30, 29, 30, 30, 26, 29 and 28 biological replicates across 2 independent experiments. **b**, *Variovorax* degrades auxin. Growth curves showing optical density at OD_600_ (top) and IAA concentrations (mg ml^−1^) (bottom) in *Variovorax* CL14 cultures grown in M9 medium with different carbon sources. Left, IAA (+ 0.5% ethanol as solvent); middle, succinate; right, succinate and IAA (+ 0.5% ethanol as solvent). **c**, *Variovorax* quenches induction of the auxin bioreporter *DR5::GFP*. Quantification of GFP intensity in *DR5::GFP Arabidopsis* seedlings grown with no bacteria, *Arthrobacter* CL28 and *Arthrobacter* CL28 + *Variovorax* CL14. GFP fluorescence was imaged 1, 3, 6, 9 and 13 d after inoculation, and quantified in the root elongation zone. Significance was determined via ANOVA within each time point, while controlling for experiment, and denoted with asterisks. *n* = 20 biological replicates across 2 independent experiments. **d**, Representative primary root images of *DR5::GFP* plants quantified in **c**, showing roots from 1, 3 and 6 d after inoculation. In addition to the bacterial treatments shown in **c**, an exogenous IAA control is shown (IAA, second column), as well as IAA-treated plants inoculated with *Variovorax* CL14, illustrating that IAA-induced fluorescence is quenched in the presence of *Variovorax* CL14 within 3 d.

**Fig. 3 |:**
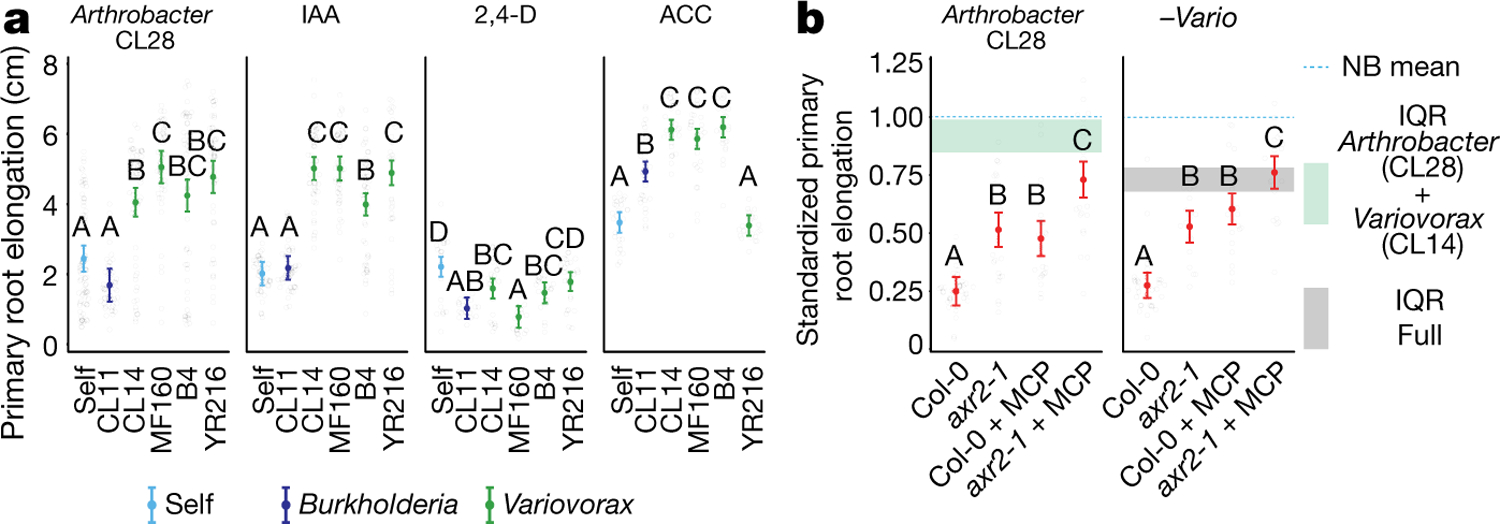
*Variovorax* suppression of RGI is related to auxin and ethylene signalling. **a**, Primary root elongation of seedlings grown with RGI-inducing *Arthrobacter* CL28 or three hormonal treatments (100 nM IAA, 2,4-dichlorophenoxyacetic acid (2,4-D) or ACC), individually (self) or with *Burkholderia* CL11 or one of four *Variovorax* isolates (CL14, MF160, B4 or YR216). Significance was determined within each treatment via ANOVA; letters correspond to a Tukey post hoc test. *n* = 74, 46, 61, 48, 49, 49, 45, 44, 46, 43, 49, 40, 22, 19, 22, 19, 20, 25, 28, 30, 29, 29, 29 and 29 (from left to right) biological replicates across 2 independent experiments. **b**, Primary root elongation, standardized to axenic conditions, of wild-type (Col-0), auxin-unresponsive (*axr2-1*), ethylene-unresponsive (Col-0 + MCP) or auxin- and ethylene-unresponsive (*axr2-1* + MCP) seedlings inoculated with RGI-inducing *Arthrobacter* CL28 or the *Variovorax* drop-out synthetic community (−*Vario*). The blue dotted line marks the relative mean length of uninoculated seedlings. Horizontal shading is the IQR of seedlings grown with *Arthrobacter* CL28 +*Variovorax* CL14 (aqua) or the full synthetic community (grey). Significance was determined via ANOVA; letters correspond to a Tukey post hoc test. *n* = 37, 25, 24, 23, 35, 21, 22 and 20 (from left to right) biological replicates across 2 independent experiments. In **a**, **b**, data represent the mean ± 95% confidence interval.

We asked whether the suppression of RGI by *Variovorax* is directly and exclusively related to auxin signalling. Besides auxin, other small molecules cause RGI. These include the plant hormones ethylene^28^ and cytokinin^29^, as well as microbial-associated molecular patterns such as the flagellin-derived peptide flg22^30^. We tested the ability of diverse *Variovorax* strains and of the *Burkholderia* strain CL11 to revert the RGI induced by auxins (indole-3-acetic acid (IAA) and the auxin analogue 2,4-dichlorophenoxyacetic acid), ethylene (the ethylene precursor 1-aminocyclopropane-1-carboxylic acid (ACC)), cytokinins (zeatin and 6-benzylaminopurine) and the flg22 peptide. All of the *Variovorax* strains we tested suppressed the RGI induced by IAA or ACC (Fig. 3a)—with the exception of *Variovorax* YR216, which did not suppress ACC-induced RGI and does not contain an ACC deaminase gene, a plant-growth-promoting feature that is associated with this genus^28^ (Extended Data Fig. 5a). *Burkholderia* CL11 only partially reverted ACC-induced RGI (Fig. 3a). None of the *Variovorax* strains attenuated the RGI induced by 2,4-dichlorophenoxyacetic acid, flg22 or cytokinins (Fig. 3a, Extended Data Fig. 8a). Importantly, this function is mediated by recognition of auxin by *Variovorax* and not by the plant auxin response per se, as the auxin response (RGI) induced by 2,4-dichlorophenoxyacetic acid is not reverted. Indeed, we found that *Variovorax* CL14 degrades IAA in culture (Extended Data Fig. 8b) and quenches fluorescence of the *Arabidopsis* auxin reporter line *DR5::GFP* caused by the RGI-inducing *Arthrobacter* CL28 (Extended Data Fig. 8c, d).

Auxin and ethylene are known to act synergistically to inhibit root growth^31^. To ascertain the roles of both auxin and ethylene perception by the plant in responding to RGI-inducing strains, we used the auxin-insensitive *axr2-1* mutant^32^ combined with a competitive inhibitor of ethylene receptors, 1-methylcyclopropene (1-MCP)^33^. We inoculated wild-type seedlings and *axr2-1* mutants, treated or not with 1-MCP, with the RGI-inducing *Arthrobacter* CL28 strain or the *Variovorax* drop-out synthetic community. We observed in both cases that bacterial RGI is reduced in *axr2-1* and 1-MCP-treated wild-type seedlings, and is further reduced in doubly insensitive 1-MCP-treated *axr2-1* seedlings; this demonstrates that both auxin and ethylene perception in the plant contribute additively to bacterially induced RGI (Fig. 3b). Thus, in the absence of *Variovorax*, a complex synthetic community can induce severe morphological changes in root phenotypes via both auxin- and ethylene-dependent pathways, but both are reverted when *Variovorax* is present.

**Extended Data Fig. 9 |:**
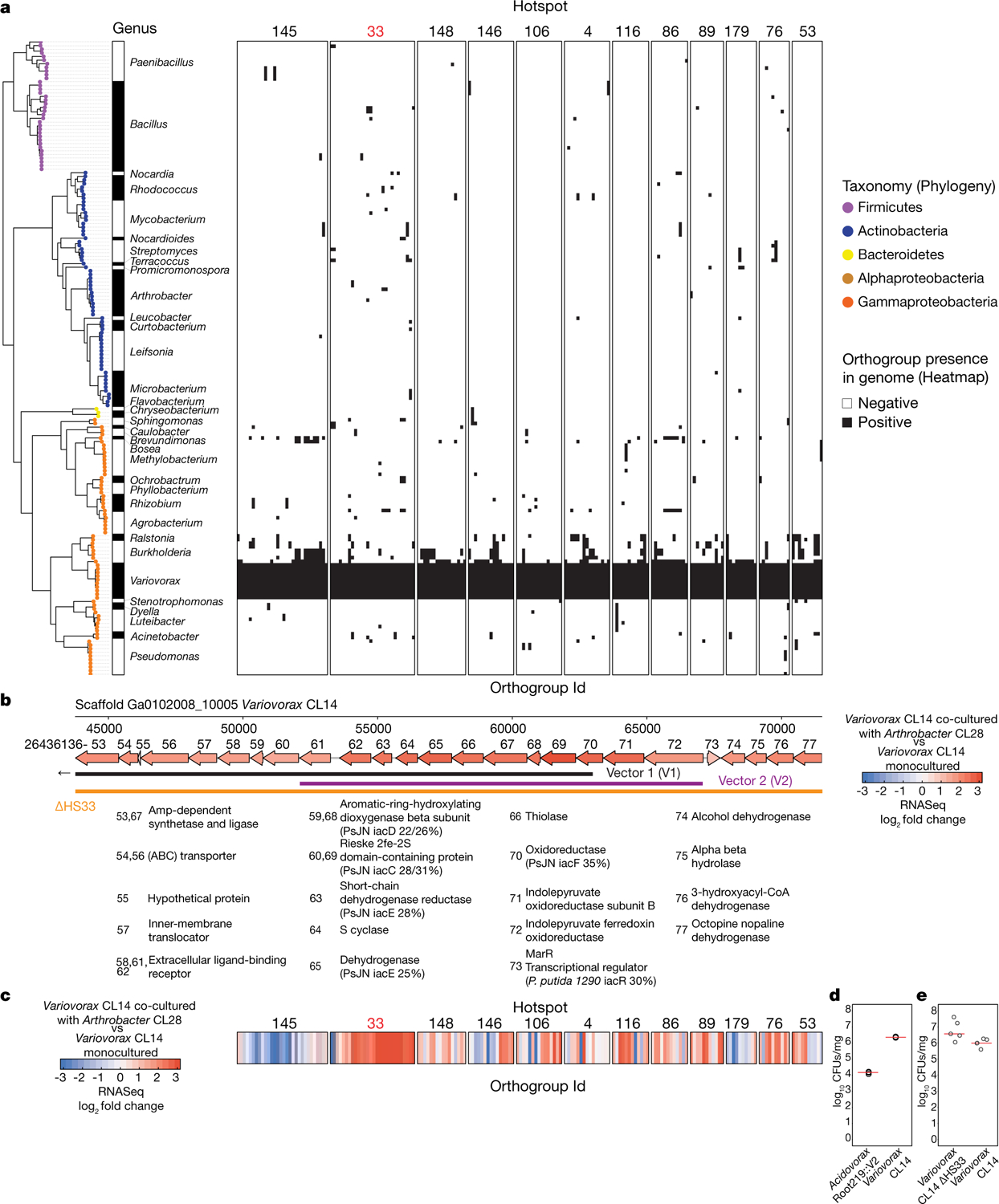
Detection of CL28-responsive *Variovorax*-unique operons. **a**, Presence–absence matrix denoting the distribution of 12 *Variovorax*-unique hotspots containing at least 10 genes across the 185 members of the synthetic community. Hotspots are defined using the *Variovorax* CL14 genome as a reference. Phylogeny of the 185 members of the synthetic community is shown to the left of the matrix. We determined the presence of an orthogroup based on a hidden Markov model profile scanning of each core *Variovorax* (genus) orthogroup across the 185 genomes in the synthetic community. **b**, A map of the auxin-degrading hotspot 33. Genes are annotated with the last two digits of their IMG gene identifier (26436136XX) and their functional assignments are shown below the map, including per cent identity of any to genes from a known auxin degradation locus. Genes are coloured by the log_2_-transformed fold change in their transcript abundance in *Variovorax* CL14 cocultured with *Arthrobacter* CL28 versus *Variovorax* CL14 monoculture, as measured by RNA-seq (shown in **c**). The overlap of this region with vectors 1 and 2 and the region knocked out in *Variovorax* CL14 ΔHS33 are shown below the map. Vector 1 extends beyond this region. **c**, Results of RNA-seq on *Variovorax* CL14 transcripts. *Variovorax* CL14 was cocultured with *Arthrobacter* CL28 versus *Variovorax* CL14 monoculture. Only *Variovorax-* unique genomic hotspots are presented, aligned with the genes in **a**. Genes are coloured by the log_2_-transformed fold change in their transcript abundance in *Variovorax* CL14 cocultured with *Arthrobacter* CL28 vs *Variovorax* CL14 monoculture. Note uniform upregulation of genes in cluster 33. **d**, log-transformed CFUs of *Variovorax* CL14 or with the *Acidovorax* gain-of-function strain *Acidovorax* root219::V2 normalized to root weight. Each of these two strains was co-inoculated with *Arthrobacter* CL28 onto 7-d-old seedlings and collected after 12 d of growth. CFU counts were determined on LB plates containing 100 μg ml^−1^ ampicillin, for which *Arthrobacter* CL28 is susceptible and *Variovorax* CL14 and *Acidovorax* root219::V2 are naturally resistant. *n* = 3 biologically independent samples. Significance was determined using a two-sided Student’s *t*-test. *P* = 2.57 × 10^−5^. Line represents median. **e**, log-transformed CFUs of *Variovorax* CL14 or of the loss-of-function strain *Variovorax* CL14 ΔHS33 normalized to root weight. Each of these two strains was inoculated individually onto 7-d-old seedlings and collected after 12 d of growth. CFU counts were determined on LB plates. *n* = 5 biologically independent samples. Significance was determined using a two-sided Student’s *t*-test. *P* = 0.049 (mutant is slightly higher). Red lines represent median.

**Fig. 4 |:**
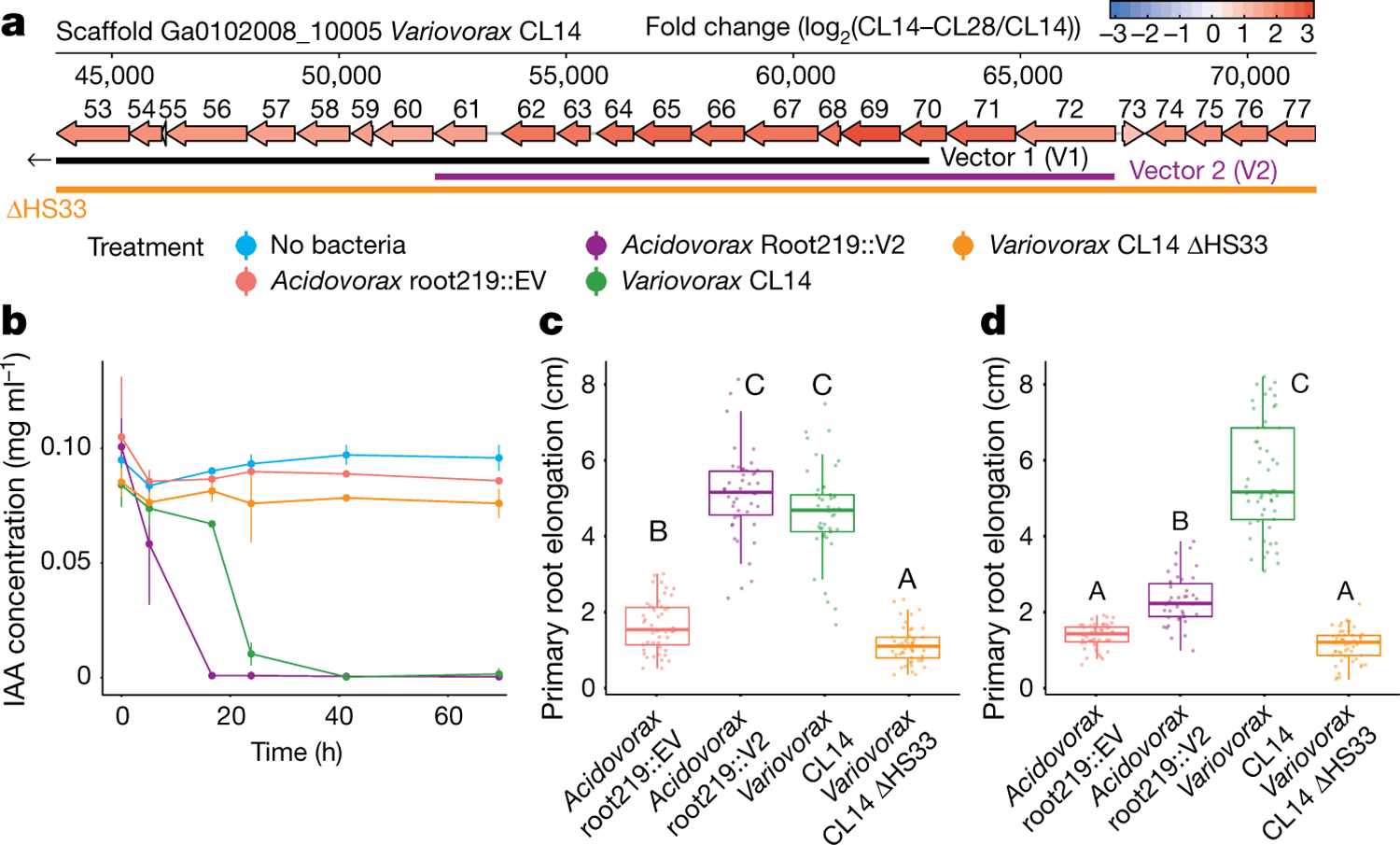
An auxin-degrading operon in *Variovorax* is required for root development. **a**, A map of the auxin-degrading hotspot 33. Genes are annotated with the last two digits of their IMG gene identifier (26436136XX) (Extended Data Fig. 9b), and are coloured by the log_2_-transformed fold change in their transcript abundance in *Variovorax* CL14 cocultured with *Arthrobacter* CL28 (CL14–CL28) relative to *Variovorax* CL14 (CL14) in monoculture (measured by RNA-seq). Genes contained in vector 1 (V1) and vector 2 (V2), and the region knocked-out in *Variovorax* CL14 ΔHS33, are shown below the map. **b**, In vitro degradation of IAA by *Acidovorax* root219::EV, *Acidovorax* Root219::V2, *Variovorax* CL14 and *Variovorax* CL14 ΔHS33. *n* = 3 biological replicates. **c**, **d**, Primary root elongation of seedlings treated with IAA (**c**) or co-inoculated with *Arthrobacter* CL28 combined with *Acidovorax* root219::EV, *Acidovorax* root219::V2, *Variovorax* CL14 and *Variovorax* CL14 ΔHS33 (**d**). Significance was determined via ANOVA; letters correspond to a Tukey post hoc test. **c**, *n* = 49, 46, 46 and 49 (from left to right); **d**, *n* = 51, 41, 52 and 53 (from left to right) biological replicates across 2 independent experiments.

To identify the bacterial mechanism or mechanisms that are involved in the attenuation of RGI, we compared the genomes of the 10 *Variovorax* strains in the synthetic community to the genomes of the other 175 members of the synthetic community. Using de novo orthologue clustering across all 185 genomes, we identified 947 genes unique to *Variovorax*, with <5% prevalence across the 175 non-*Variovorax* members of the synthetic community and 100% prevalence among all 10 *Variovorax* strains. We grouped these genes into regions of physically contiguous genes (genomic hotspots) and focused on the 12 hotspots that contained at least 10 genes unique to *Variovorax* (Extended Data Fig. 9a, Supplementary Table 3). One of these hotspots (designated hotspot 33) contains weak homologues (average of about 30% identity) to the genes *iacC*, *iacD*, *iacE*, *iacF* and *iacR* of the IAA-degrading *iac* operon of *Paraburkholderia phytofirmans* strain PsJN^18^, but lacks *iacA*, *iacB* and *iacI*—which are known to be necessary for *Paraburkholderia* growth on IAA^17^ (Fig. 4a, Extended Data Fig. 9b). To test whether the hotspots we identified are responsive to RGI-inducing bacteria, we analysed the transcriptome of *Variovorax* CL14 in monoculture and in coculture with the RGI-inducing *Arthrobacter* CL28. We observed extensive transcriptional reprogramming of *Variovorax* CL14 when cocultured with *Arthrobacter* CL28 (Supplementary Table 4). Among the 12 hotspots we identified, the genes in hotspot 33 were the most highly upregulated (Fig. 4a, Extended Data Fig. 9b, c). We thus hypothesized that hotspot 33 contains an uncharacterized auxin-degradation operon.

In parallel, we constructed a *Variovorax* CL14 genomic library in *Escherichia coli* with >12.5-kb inserts in a broad host-range vector, and screened the resulting *E. coli* clones for auxin degradation. Two clones from the approximately 3,500 that we screened degraded IAA (denoted V1 and V2) (Supplementary Table 5). The *Variovorax* CL14 genomic inserts in both of these clones contained portions of hotspot 33 (Fig. 4a, Extended Data Fig. 9b). The overlap common to both of these clones contained nine genes, among them the weak homologues to *Paraburkholedria iacC*, *iacD* and *iacE*. To test whether this genomic region is sufficient to revert RGI in plants, we transformed *Acidovorax* root219, a relative of *Variovorax* that does not cause or revert RGI (Extended Data Fig. 5a, b), with the shorter functional insert (V2) (Fig. 4a, Extended Data Fig. 9b) or with an empty vector (EV). The resulting gain-of-function strain *Acidovorax* root219::V2 gained the ability to degrade IAA in culture (Fig. 4b). We inoculated *Acidovorax* root219::V2 or the control *Acidovorax* root219::EV onto plants treated with IAA or inoculated with the RGI-inducing *Arthrobacter* CL28. *Acidovorax* root219::V2 fully reverted IAA-induced RGI (Fig. 4c) and partially reverted *Arthrobacter* CL28-induced RGI, despite colonizing roots at significantly lower levels than *Variovorax* CL14 (Fig. 4d, Extended Data Fig. 9d). In addition, we deleted hotspot 33 from *Variovorax* CL14 (Fig. 4a) to test whether this putative operon is necessary for the reversion of RGI. The resulting strain *Variovorax* CL14 ΔHS33—which is not impaired in plant colonization (Extended Data Fig. 9e)—did not degrade IAA in culture (Fig. 4b), and did not revert IAA-induced (Fig. 4c) or *Arthrobacter* CL28-induced RGI (Fig. 4d). Thus, this *Variovorax*-specific gene cluster is necessary for the suppression of RGI and for auxin degradation. It is thus the critical genetic locus required by *Variovorax* to maintain stereotypic root development in the context of a phylogenetically diverse microbiome.

## Conclusions

Signalling molecules and other secondary metabolites are products of adaptations that allow microorganisms to survive competition for primary metabolites. Our results illuminate the importance of a trophic layer of microorganisms that use these secondary metabolites for their own benefit, while potentially providing the unselected exaptation^34^ of interfering with signalling between the bacterial microbiota and the plant host. Such metabolic signal interference has previously been demonstrated in the case of quorum quenching^12^, the degradation of microbial-associated molecular patterns^35^ and the degradation of bacterially produced auxin, including among *Variovorax*^13–15^. Plant development relies on tightly regulated auxin concentration gradients^19^, which can be distorted by auxin fluxes emanating from the microbiota. Some *Variovorax* strains have the capacity to both produce and degrade auxin, which suggests a capacity to fine-tune auxin concentrations in the rhizosphere^15,28^.

**Extended Data Fig. 10 |:**
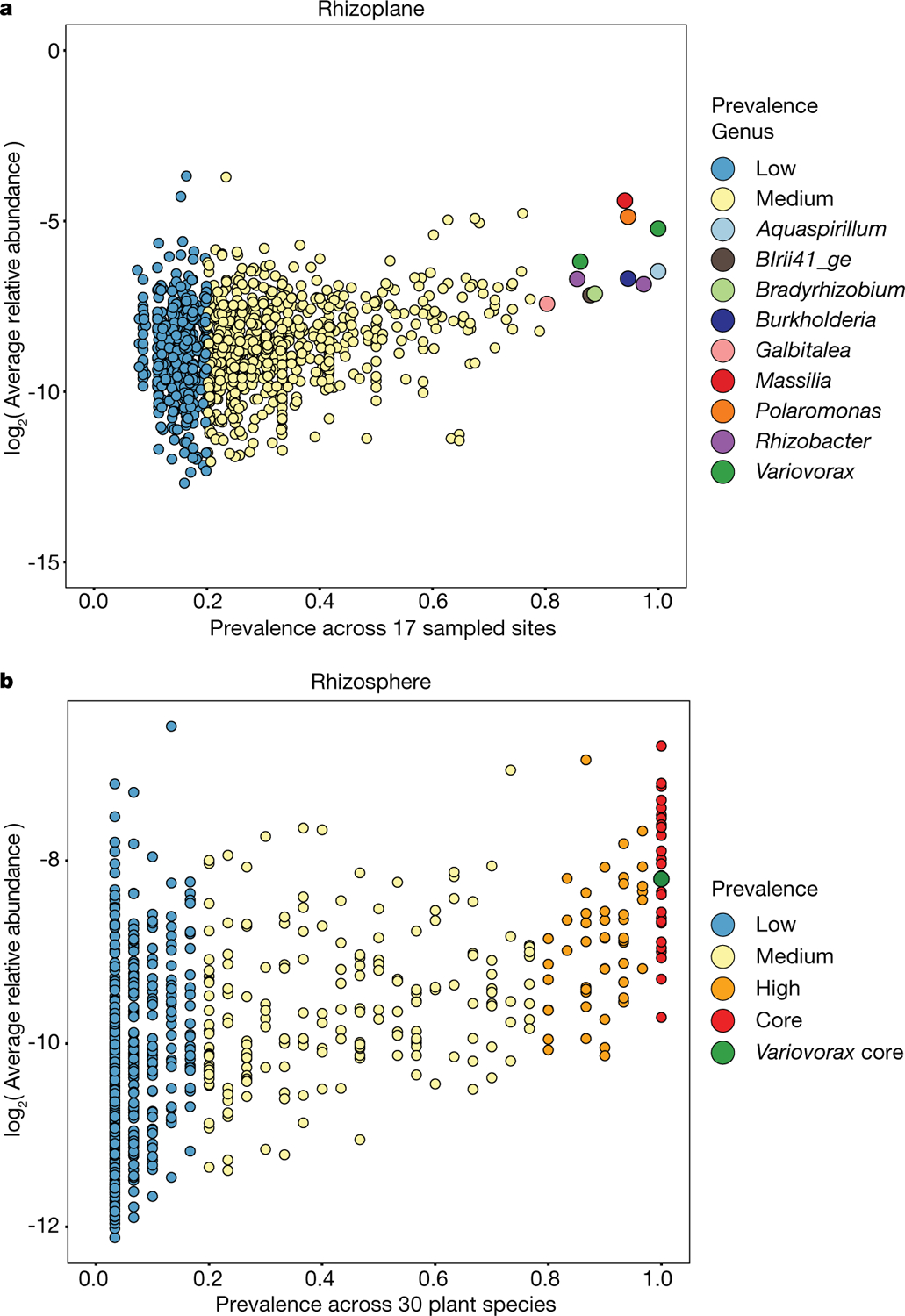
*Variovorax* are highly prevalent across naturally occurring *Arabidopsis* microbiomes and across 30 plant species. **a**, Correlation plot of data reanalysed from ref.^6^, comparing bacterial ASV prevalence to log-transformed relative abundance in *A. thaliana* rhizoplane samples taken across 3 years in 17 sites in Europe. **b**, Correlation plot of data reanalysed from ref.^4^, comparing bacterial ASV prevalence to log-transformed relative abundance across 30 phylogenetically diverse plant species grown in a common garden experiment.

We have shown here that the chemical homeostasis enforced by the presence of *Variovorax* in a phylogenetically diverse, realistic synthetic community allows the plant to maintain its developmental programme within a chemically complex matrix. *Variovorax* was recently found to have the rare property of improved plant colonization upon late arrival to an established community^10^, which suggests that they use bacterially produced or induced—rather than plant-derived—substrates. Furthermore, after re-analysing a recent large-scale time and spatially resolved survey of the *Arabidopsis* root microbiome^6^ and a common garden experiment including 30 plant species^4^, we noted that *Variovorax* is among a limited group of core bacterial genera found in 100% of the sampled sites and plant species (Extended Data Fig. 10a, b). These ecological observations, together with our results using a reductionist microcosm, reinforce the importance of *Variovorax* as a key player in the bacteria–bacteria–plant communication networks that are required to maintain root growth within a complex biochemical ecosystem.

## Methods

No statistical methods were used to predetermine sample size. The experiments were randomized, and investigators were not blinded to allocation during experiments and outcome assessment.

### *Arabidopsis* with bacterial synthetic community microcosm across four stress gradients

This section relates to Fig. 1a, Extended Data Fig. 1.

#### Bacterial culture and plant inoculation.

The 185-member bacterial synthetic community used here contains genome-sequenced isolates obtained from surface-sterilized Brassicaceae roots, nearly all *Arabidopsis thaliana*, planted in two soils from North Carolina (USA). A detailed description of this collection and isolation procedures can be found in ref.^20^. One week before each experiment, bacteria were inoculated from glycerol stocks into 600 μl KB medium in a 96-deep-well plate. Bacterial cultures were grown at 28 °C, shaking at 250 rpm. After 5 days of growth, cultures were inoculated into fresh medium and returned to the incubator for an additional 48 h, resulting in 2 copies of each culture (7 days old and 48 h old). We adopted this procedure to account for variable growth rates of different members of the synthetic community and to ensure that nonstationary cells from each strain were included in the inoculum. After growth, 48-h and 7-day plates were combined and optical density of cultures was measured at 600 nm (OD_600_) using an Infinite M200 Pro plate reader (TECAN). All cultures were then pooled while normalizing the volume of each culture to OD_600_ = 1. The mixed culture was washed twice with 10 mM MgCl_2_ to remove spent medium and cell debris, and vortexed vigorously with sterile glass beads to break up aggregates. OD_600_ of the mixed, washed culture was then measured and normalized to OD_600_ = 0.2. The synthetic community inoculum (100 μl) was spread on 12 × 12-cm vertical square agar plates with amended Johnson medium (JM)^1^ without sucrose before transferring seedlings.

#### In vitro plant growth conditions.

All seeds were surface-sterilized with 70% bleach, 0.2% Tween-20 for 8 min, and rinsed 3 times with sterile distilled water to eliminate any seed-borne microorganisms on the seed surface. Seeds were stratified at 4 °C in the dark for 2 days. Plants were germinated on vertical square 12 × 12-cm agar plates with JM containing 0.5% sucrose, for 7 days. Then, 10 plants were transferred to each of the agar plates inoculated with the synthetic community. The composition of JM in the agar plates was amended to produce environmental variation. We added to the previously reported phosphate concentration gradient (0, 10, 30, 50, 100 and 1,000 μM Pi)^5^ three additional environmental gradients: salinity (50, 100, 150 and 200 mM NaCl), pH (5.5, 7.0 and 8.2) and incubation temperature (10, 21 and 31 °C). Each gradient was tested separately, in two independent replicas. Each condition included three synthetic community + plant samples, two no-plant controls and one no-bacteria control. Thus, the total sample size for each condition was *n* = 6. Previous publications^1,3,5^ have shown that an *n* ≥ 5 is provides sufficient power for synthetic community profiling. Plates were placed in randomized order in growth chambers and grown under a 16-h dark/8-h light regime at 21-°C day/18-°C night for 12 days. Upon collection, DNA was extracted from roots, shoots and the agar substrate. Here and hereafter, all measurements were taken from distinct samples.

#### DNA extraction.

Roots, shoots and agar were collected separately, pooling 6–8 plants for each sample. Roots and shoots were placed in 2-ml Eppendorf tubes with 3 sterile glass beads. These samples were washed three times with sterile distilled water to remove agar particles and weakly associated microorganisms. Tubes containing the samples were stored at −80 °C until processing. Root and shoot samples were lyophilized for 48 h using a Labconco freeze-dry system and pulverized using a tissue homogenizer (MPBio). Agar from each plate was collected in 30-ml syringes with a square of sterilized Miracloth (Millipore) at the bottom and kept at −20 °C for 1 week. Syringes were then thawed at room temperature and samples were squeezed gently through the Miracloth into 50-ml falcon tubes. Samples were centrifuged at maximum speed for 20 min and most of the supernatant was discarded. The remaining 1–2 ml of supernatant, containing the pellet, was transferred into clean 1.5-ml Eppendorf tubes. Samples were centrifuged again, supernatant was removed and pellets were stored at −80 °C until DNA extraction. DNA extractions were carried out on ground root and shoot tissue and agar pellets using 96-well-format MoBio PowerSoil Kit (MOBIO Laboratories; Qiagen) following the manufacturer’s instruction. Sample position in the DNA extraction plates was randomized, and this randomized distribution was maintained throughout library preparation and sequencing.

#### Bacterial 16S rRNA sequencing.

We amplified the V3–V4 regions of the bacterial 16S rRNA gene using the primers 338F (5′-ACTCCTACGGGA GGCAGCA-3′) and 806R (5′-GGACTACHVGGGTWTCTAAT-3′). Two barcodes and six frameshifts were added to the 5′ end of 338F and six frameshifts were added to the 806R primers, on the basis of a previously published protocol^36^. Each PCR reaction was performed in triplicate, and included a unique mixture of three frameshifted primer combinations for each plate. PCR conditions were as follows: 5 μl Kapa Enhancer, 5 μl Kapa Buffer A, 1.25 μl 5 μM 338F, 1.25 μl 5 μM 806R, 0.375 μl mixed plant rRNA gene-blocking peptide nucleic acids (PNAs) (1:1 mix of 100 μM plastid PNA and 100 μM mitochondrial PNA^36^), 0.5 μl Kapa dNTPs, 0.2 μl Kapa Robust Taq, 8 μl dH_2_O, 5 μl DNA; temperature cycling: 95 °C for 60 s; 24 cycles of 95 °C for 15 s; 78 °C (PNA) for 10 s; 50 °C for 30 s; 72 °C for 30 s; 4 °C until use. Following PCR clean up, using AMPure beads (Beckman Coulter), the PCR product was indexed using 96 indexed 806R primers with the Kapa HiFi Hotstart readymix with the same primers as above; temperature cycling: 95 °C for 60 s; 9 cycles of 95 °C for 15 s; 78 °C (PNA) for 10 s; 60 °C for 30 s; 72 °C for 35 s; 4 °C until use. PCR products were purified using AMPure XP magnetic beads (Beckman Coulter) and quantified with a Qubit 2.0 fluorometer (Invitrogen). Amplicons were pooled in equal amounts and then diluted to 10 pM for sequencing. Sequencing was performed on an Illumina MiSeq instrument using a 600-cycle V3 chemistry kit. DNA sequence data for this experiment are available at the NCBI Bioproject repository (accession PRJNA543313). The abundance matrix, metadata and taxonomy are available at https://github.com/isaisg/variovoraxRGI.

#### 16S rRNA amplicon sequence data processing.

Synthetic community sequencing data were processed with MT-Toolbox^37^. Usable read output from MT-Toolbox (that is, reads with 100% correct primer and primer sequences that successfully merged with their pair) were quality-filtered using Sickle^38^ by not allowing any window with *Q* score under 20. The resulting sequences were globally aligned to a reference set of 16S rDNA sequences extracted from genome assemblies of members of the synthetic community. For strains that did not have an intact 16S rDNA sequence in their assembly, we sequenced the 16S rRNA gene using Sanger sequencing. The reference database also included sequences from known bacterial contaminants and *Arabidopsis* organellar sequences. Sequence alignment was performed with USEARCH v.7.1090^39^ with the option usearch_global at a 98% identity threshold. On average, 85% of sequences matched an expected isolate. Our 185 isolates could not all be distinguished from each other based on the V3–V4 sequence and were thus classified into 97 unique sequences. A unique sequence encompasses a set of identical (clustered at 100%) V3–V4 sequences coming from a single or multiple isolates.

Sequence mapping results were used to produce an isolate abundance table. The remaining unmapped sequences were clustered into operational taxonomic units (OTUs) using UPARSE^40^ implemented with USEARCH v.7.1090, at 97% identity. Representative OTU sequences were taxonomically annotated with the RDP classifier^41^ trained on the Greengenes database^42^ (4 February 2011). Matches to *Arabidopsis* organelles were discarded. The vast majority of the remaining unassigned OTUs belonged to the same families as isolates in the synthetic community. We combined the assigned unique sequence and unassigned OTU count tables into a single count table. In addition to the raw count table, we created rarefied (1,000 reads per sample) and relative abundance versions of the abundance matrix for further analyses.

The resulting abundance tables were processed and analysed with functions from the ohchibi package (https://github.com/isaisg/ohchibi). An α-diversity metric (Shannon diversity) was calculated using the diversity function from the vegan package v.2.5–3^43^. We used ANOVA to test for differences in α-diversity between groups. β-Diversity analyses (principal coordinate analysis and canonical analysis of principal coordinates (CAP)) were based on Bray–Curtis dissimilarity calculated from the relative abundance matrices. We used the capscale function from the vegan R package v.2.5–3^43^ to compute the CAP. To analyse the full dataset (all fractions and all abiotic treatments), we constrained by fraction and abiotic treatment while conditioning for the replica and experiment effect. We explored the abiotic conditions effect inside each of the four abiotic gradients tested (phosphate, salinity, pH and temperature). We performed the fraction–abiotic interaction analysis within each fraction independently, constraining for the abiotic conditions while conditioning for the replica effect. In addition to CAP, we performed PERMANOVA using the adonis function from the vegan package v.2.5–3^43^. We used the package DESeq2 v.1.22.1^44^ to compute the enrichment profiles for unique sequences present in the count table.

We estimated the fraction effect across all the abiotic conditions tested by creating a group variable that merged the fraction variable and the abiotic condition variable together (for example, Root_10Pi, Substrate_10Pi). We fitted the following model specification using this group variable: abundance ~ rep + experiment + group.

From the fitted model, we extracted—for all levels within the group variables—the following comparisons: substrate versus root and substrate versus shoot. A unique sequence was considered statistically significant if it had a FDR-adjusted *P* value < 0.05.

All scripts and dataset objects necessary to reproduce the synthetic community analyses are deposited in the following GitHub repository: https://github.com/isaisg/variovoraxRGI.

#### Co-occurrence analysis.

The relative abundance matrix (unique sequences × samples) was standardized across the unique sequences by dividing the abundance of each unique sequence in its sample over the mean abundance of that unique sequence across all samples. Subsequently, we created a dissimilarity matrix based on the Pearson correlation coefficient between all the pairs of strains in the transformed abundance matrix, using the cor function in the stats base package in R. Finally, hierarchichal clustering (method ward.D2, function hclust) was applied over the dissimilarity matrix constructed above.

#### Heat map and family enrichment analysis.

We visualized the results of the generalized linear model (GLM) testing the fraction effects across each specific abiotic condition tested using a heat map. The rows in the heat map were ordered according to the dendrogram order obtained from the unique sequences co-occurrence analysis. The heat map was coloured on the basis of the log_2_-transformed fold change output by the GLM model. We highlighted in a black shade the comparisons that were significant (*q* value < 0.05). Finally, for each of the four modules, we computed for each family present in that module a hypergeometric test testing if that family was overrepresented (enriched) in that particular module. Families with an FDR-adjusted *P* value < 0.1 are visualized in the figure.

### Deconstructing the synthetic community to four modules of co-occurring strains

This section relates to Fig. 1a–c.

#### Bacterial culture and plant-inoculation.

Strains belonging to each module A, B, C and D (‘Co-occurrence analysis’ in ‘*Arabidopsis* with bacterial synthetic community microcosm across four stress gradients’) were grown in separate deep 96-well plates and mixed as described in ‘Bacterial culture and plant-inoculation’ in ‘*Arabidopsis* with bacterial synthetic community microcosm across four stress gradients’. The concentration of each module was adjusted to OD_600_ = 0.05 (1/4 of the concentration of the full synthetic community). Each module was spread on the plates either separately, or in combination with another module at a total volume of 100 μl. In addition, we included a full synthetic community control and an uninoculated control, bringing the number of synthetic community combinations to 12. We performed the experiment in two independent replicates and each replicate included five plates per synthetic community combination.

#### In vitro plant growth conditions.

Seed sterilization and germination conditions were the same as in ‘In vitro plant growth conditions’ in ‘*Arabidopsis* with bacterial synthetic community microcosm across four stress gradients’. Plants were transferred to each of the synthetic-community-inoculated agar plates containing JM without sucrose. Plates were placed in randomized order in growth chambers and grown under a 16-h dark/8-h light regime at 21-°C day/18-°C night for 12 days. Upon collection, root morphology was measured.

#### Root and shoot image analysis.

Plates were imaged 12 days after transfer, using a document scanner. Primary root length elongation was measured using ImageJ^45^ and shoot area and total root network were measured with WinRhizo software (Regent Instruments).

#### Primary root elongation analyses.

Primary root elongation was compared across the no bacteria, full synthetic community, single modules and pairs of modules treatments jointly using a two-sided ANOVA model controlling for the replicate effect. We inspected the normality assumptions (here and elsewhere) using qqplots and Shapiro tests. Differences between treatments were indicated using the confidence letter display derived from the Tukey’s post hoc test implemented in the package emmeans^46^.

### Inoculating plants with all synthetic community isolates separately

This section relates to Extended Data Fig. 2a–c.

#### Bacterial culture and plant inoculation.

Cultures from each strain in the synthetic community were grown in KB medium and washed separately (‘Bacterial culture and plant inoculation’ in ‘*Arabidopsis* with bacterial synthetic community microcosm across four stress gradients’), and OD_600_ was adjusted to 0.01 before spreading 100 μl on plates. We performed the experiment in two independent replicates and each replicate included one plate per each of the 185 strains. In vitro growth conditions were the same as described in ‘In vitro plant growth conditions’ in ‘Deconstructing the synthetic community to four modules of co-occurring strains’. Upon collection, root morphology was measured (‘Root and shoot image analysis’ in ‘Deconstructing the synthetic community to four modules of co-occurring strains’). Isolates generating an average main root elongation of <3 cm were classified as RGI-inducing strains.

### Tripartite plant–microorganism–microorganism experiments

This section relates to Fig. 1d, e, Extended Data Fig. 3a.

#### Experimental design.

To identify strains that revert RGI (Fig. 1d), we selected all 18 non-RGI-inducing strains in module A and co-inoculated them with each of four RGI-inducing strains, one from each module. The experiment also included uninoculated controls and controls consisting of each of the 22 strains inoculated alone, amounting to 95 separate bacterial combinations.

To confirm the ability of *Variovorax* and *Burkholderia* to attenuate RGI induced by diverse bacteria (Extended Data Fig. 3a), three RGI-suppressing strains were co-inoculated with a selection of 18 RGI-inducing strains. The experiment also included uninoculated controls and controls consisting of each of the 21 strains inoculated alone. Thus, the experiment consisted of 76 separate bacterial combinations. We performed each of these two experiments in two independent replicates and each replicate included one plate per each of the strain combinations.

#### Bacterial culture and plant-inoculation.

All strains were streaked on agar plates, then transferred to 4 ml liquid KB medium for over-night growth. Cultures were then washed, and OD_600_ was adjusted to 0.02 before mixing and spreading 100 μl on each plate. Upon collection, root morphology was measured (‘Root and shoot image analysis’ in ‘Deconstructing the synthetic community to four modules of co-occurring strains’) and plant RNA was collected and processed from uninoculated samples, and from samples with *Variovorax* CL14, the RGI-inducing strain *Arthrobacter* CL28 and the combination of both (‘Experimental design’ in ‘Tripartite plant–microorganism–microorganism experiments’).

#### Primary root elongation analysis.

We fitted ANOVA models for each RGI-inducing strain we tested. Each model compared the primary root elongation with the RGI-inducing strains alone against root elongation when the RGI-inducing strain was co-inoculated with other isolates. The *P* values for all the comparisons were corrected for multiple testing using FDR.

#### RNA extraction.

RNA was extracted from *A. thaliana* seedlings following previously published methods^47^. Four seedlings were pooled from each plate and 3–5 samples per treatment were flash frozen and stored at −80 °C until processing. Frozen seedlings were ground using a TissueLyzer II (Qiagen), then homogenized in a buffer containing 400 μl of Z6-buffer; 8 M guanidine HCl, 20 mM MES, 20 mM EDTA at pH 7.0. Four hundred μl phenol:chloroform:isoamylalcohol, 25:24:1 was added, and samples were vortexed and centrifuged (20,000*g*, 10 min) for phase separation. The aqueous phase was transferred to a new 1.5-ml Eppendorf tube and 0.05 volumes of 1 N acetic acid and 0.7 volumes 96% ethanol were added. The RNA was precipitated at −20 °C overnight. Following centrifugation (20,000*g*, 10 min, 4 °C), the pellet was washed with 200 μl sodium acetate (pH 5.2) and 70% ethanol. The RNA was dried and dissolved in 30 μl of ultrapure water and stored at −80 °C until use.

#### Plant RNA sequencing.

Illumina-based mRNA-seq libraries were prepared from 1 μg RNA following previously published methods^3^. mRNA was purified from total RNA using Sera-mag oligo(dT) magnetic beads (GE Healthcare Life Sciences) and then fragmented in the presence of divalent cations (Mg^2+^) at 94 °C for 6 min. The resulting fragmented mRNA was used for first-strand cDNA synthesis using random hexamers and reverse transcriptase, followed by second-strand cDNA synthesis using DNA polymerase I and RNaseH. Double-stranded cDNA was end-repaired using T4 DNA polymerase, T4 polynucleotide kinase and Klenow polymerase. The DNA fragments were then adenylated using Klenow exo-polymerase to allow the ligation of Illumina Truseq HT adapters (D501–D508 and D701–D712). All enzymes were purchased from Enzymatics. Following library preparation, quality control and quantification were performed using a 2100 Bioanalyzer instrument (Agilent) and the Quant-iT PicoGreen dsDNA Reagent (Invitrogen), respectively. Libraries were sequenced using Illumina HiSeq4000 sequencers to generate 50-bp single-end reads.

#### RNA-seq read processing.

Initial quality assessment of the Illumina RNA-seq reads was performed using FastQC v.0.11.7 (Babraham Bioinformatics). Trimmomatic v.0.36^48^ was used to identify and discard reads containing the Illumina adaptor sequence. The resulting high-quality reads were then mapped against the TAIR10 *Arabidopsis* reference genome using HISAT2 v.2.1.0^49^ with default parameters. The featureCounts function from the Subread package^50^ was then used to count reads that mapped to each one of the 27,206 nuclear protein-coding genes. Evaluation of the results of each step of the analysis was performed using MultiQC v.1.1^51^. Raw sequencing data and read counts are available at the NCBI Gene Expression Omnibus accession number GSE131158.

### *Variovorax* drop-out experiment

This section relates to Fig. 2a–c, g, h, Extended Data Fig. 4a.

#### Bacterial culture and plant-inoculation.

The entire synthetic community, excluding all 10 *Variovorax* isolates and all 5 *Burkholderia* isolates, was grown and prepared as described in ‘Bacterial culture and plant inoculation’ in ‘*Arabidopsis* with bacterial synthetic community microcosm across four stress gradients’. The *Variovorax* and *Burkholderia* isolates were grown in separate tubes, washed and added to the rest of the synthetic community to a final OD_600_ of 0.001 (the calculated OD_600_ of each individual strain in a 185-member synthetic community at a total of OD_600_ of 0.2), to form the following five mixtures: (i) full community: all *Variovorax* and *Burkholderia* isolates added to the synthetic community; (ii) *Burkholderia* drop-out: only *Variovorax* isolates added to the synthetic community; (iii) *Variovorax* drop-out: only *Burkholderia* isolates added to the synthetic community; (iv) *Variovorax* and *Burkholderia* drop-out: no isolates added to the synthetic community; (v) uninoculated plants: no synthetic community. The experiment consisted of six plates per synthetic community mixture, amounting to 30 plates. Upon collection, root morphology was measured and analysed (‘Root and shoot image analysis’ in ‘Deconstructing the synthetic community to four modules of co-occurring strains’, and in ‘Primary root elongation analysis’ in ‘Tripartite plant–microorganism–microorganism experiments’); and bacterial DNA (‘DNA extraction’ and ‘Bacterial 16S rRNA sequencing’ in ‘*Arabidopsis* with bacterial synthetic community microcosm across four stress gradients’) and plant RNA (‘RNA extraction’ and ‘Plant RNA sequencing’ in ‘Tripartite plant–microorganism–microorganism experiments’) were collected and processed.

### *Variovorax* drop-out under varying abiotic contexts

This section relates to Fig. 2d, f, Extended Data Fig. 4c, d.

#### Bacterial culture and plant-inoculation.

The composition of JM in the agar plates was amended to produce abiotic environmental variation. These amendments included salt stress (150 mM NaCl), low phosphate (10 μM phosphate), high pH (pH 8.2) and high temperature (plates incubated at 31 °C), as well as an unamended JM control. Additionally, we tested a different medium (1/2-strength Murashige and Skoog (MS)) and a soil-like substrate. As a soil-like substrate, we used calcined clay (Diamond Pro), prepared as follows: 100 ml of clay was placed in Magenta GA7 jars. The jars were then autoclaved twice. Forty ml of liquid JM was added to the Magenta jars, with the corresponding bacterial mixture spiked into the media at a final OD_600_ of 5 × 10^−4^. Four 1-week old seedlings were transferred to each vessel, and vessels were covered with Breath-Easy gas permeable sealing membrane (Research Products International) to maintain sterility and gas exchange.

The entire synthetic community, excluding all 10 *Variovorax* isolates, was grown and prepared as described in ‘Bacterial culture and plant inoculation’ in ‘*Arabidopsis* with bacterial synthetic community microcosm across four stress gradients’. The *Variovorax* isolates were grown in separate tubes, washed and added to the rest of the synthetic community to a final OD_600_ of 0.001 (the calculated OD_600_ of each individual strain in a 185-member synthetic community at an OD_600_ of 0.2), to form the following five mixtures: (i) full community: all *Variovorax* isolates added to the synthetic community; (ii) *Variovorax* drop-out: no isolates added to the synthetic community; (iii) uninoculated plants: no synthetic community.

We inoculated all 3 synthetic community combinations in all 7 abiotic treatments, amounting to 21 experimental conditions. We performed the experiment in 2 independent replicates and each replicate included 3 plates per experimental conditions, amounting to 63 plates per replicate. Upon collection, root morphology was measured (‘Root and shoot image analysis’ in ‘Deconstructing the synthetic community to four modules of co-occurring strains’); and Bacterial DNA (‘DNA extraction, ‘Bacterial 16S rRNA sequencing’ and ‘16S rRNA amplicon sequence data processing’ in ‘*Arabidopsis* with bacterial synthetic community microcosm across four stress gradients’) and plant RNA (‘RNA extraction’, ‘Plant RNA sequencing’ and ‘RNA-seq read processing’ in ‘Tripartite plant–microorganism–microorganism experiments’) were collected and processed.

#### Root image analysis.

For agar plates, roots were imaged as described in ‘Root and shoot image analysis’ in ‘Deconstructing the synthetic community to four modules of co-occurring strains’. For calcined clay pots, four weeks after transferring, pots were inverted, and whole root systems were gently separated from the clay by washing with water. Root systems were spread over an empty Petri dish and scanned using a document scanner.

#### Primary root elongation and total root network analysis.

Primary root elongation was compared between synthetic-community treatments within each of the different abiotic contexts tested independently. Differences between treatments were indicated using the confidence letter display derived from the Tukey’s post hoc test implemented in the package emmeans.

#### Bacterial 16S rRNA data analysis.

To be able to compare shifts in the community composition of samples treated with and without the *Variovorax* genus, we in silico-removed the 10 *Variovorax* isolates from the count table of samples inoculated with the full community treatment. We then merged this count table with the count table constructed from samples inoculated without the *Variovorax* genus (*Variovorax* drop-out treatment). Then, we calculated a relative abundance of each unique sequence across all the samples using the merged count matrix. Finally, we applied CAP over the merged relative abundance matrix to control for the replica effect. In addition, we used the function adonis from the vegan R package to compute a PERMANOVA test over the merged relative abundance matrix and we fitted a model evaluating the fraction and synthetic community (presence of *Variovorax*) effects over the assembly of the community.

### *Variovorax* drop-out under varying biotic contexts

This section relates to Fig. 2e, Extended Data Fig. 4b.

#### Bacterial culture and plant inoculation.

Strains belonging to modules A (excluding *Variovorax*), C and D were grown in separate wells in deep 96-well plates and mixed as described in ‘Bacterial culture and plant inoculation’ in ‘*Arabidopsis* with bacterial synthetic community microcosm across four stress gradients’. The concentration of each module was adjusted to OD_600_ = 0.05 (1/4 of the concentration of the full synthetic community). The *Variovorax* isolates were grown in separate tubes, washed and added to the rest of the synthetic community to a final OD_600_ of 0.001.

In a separate experiment, the 35-member synthetic community used in ref.^1^ was grown, excluding *Variovorax* CL14, to create a taxonomically diverse, *Variovorax*-free subset of the full 185-member community. The concentration of this synthetic community was adjusted to OD_600_ = 0.05. The *Variovorax* isolates were grown in separate tubes, washed and added to the rest of the synthetic community to a final OD_600_ of 0.001.

These two experiments included the following mixtures (Extended Data Fig. 4b): (i) module A excluding *Variovorax*; (ii) module C; (iii) module D; (iv) module A including *Variovorax*; (v) module C + all 10 *Variovorax*; (vi) module D + all 10 *Variovorax*; (vii) 35-member synthetic community excluding the one *Variovorax* found therein; (viii) 34-member synthetic community + all 10 *Variovorax*; (ix) uninoculated control. The experiment with modules A, C and D was performed in two independent experiments, with two plates per treatment in each. The experiment with the 34-member synthetic community was performed once, with 5 plates per treatment. Upon collection, root morphology was measured (‘Root and shoot image analysis’ in ‘Deconstructing the synthetic community to four modules of co-occurring strains’).

#### Primary root elongation analysis.

We directly compared differences between the full synthetic community and *Variovorax* drop-out treatment using a *t*-test and adjusting the *P* values for multiple testing using FDR.

### Phylogenetic inference of the synthetic community and *Variovorax* isolates

This section relates to Figs. 1d, 2a, Extended Data Figs. 1a, 2b, 3a, 4b, 5a, b, 9a.

To build the phylogenetic tree of the synthetic community isolates, we used the previously described super matrix approach^20^. We scanned 120 previously defined marker genes across the 185 isolate genomes from the synthetic community using the hmmsearch tool from the hmmer v.3.1b2^52^. Then, we selected 47 markers that were present as single-copy genes in 100% of our isolates. Next, we aligned each individual marker using MAFFT^53^ and filtered low-quality columns in the alignment using trimAl^54^. Then, we concatenated all filtered alignments into a super alignment. Finally, FastTree v.2.1^55^ was used to infer the phylogeny using the WAG model of evolution. For the tree of the relative of *Variovorax*, we chose 56 markers present as single copy across 124 Burkholderiales isolates and implemented the same methodology described above.

### Measuring how prevalent the RGI suppression trait is across the *Variovorax* phylogeny

This section relates to Fig. 3a, Extended Data Fig. 5a, b.

#### Bacterial culture and plant inoculation.

Fifteen *Variovorax* strains from across the phylogeny of the genus were each co-inoculated with the RGI-inducing *Arthrobacter* CL28. All 16 strains were grown in separate tubes, then washed and OD_600_ was adjusted to 0.01 before mixing. Pairs of strains were mixed in 1:1 ratios and spread at a total volume of 100 μl onto agar before seedling transfer. The experiment also included uninoculated controls and controls consisting of each of the 16 strains inoculated alone. Thus, the experiment consisted of 32 separate bacterial combinations. We performed the experiment one time, which included three plates per bacterial combination. Upon collection, primary root elongation was analysed as described in “Root and shoot image analysis’ in ‘Deconstructing the synthetic community to four modules of co-occurring strains’.

### Measuring RGI in tomato seedlings

This section relates to Extended Data Fig. 3b.

#### Experimental design.

This experiment included the following treatments: (i) no bacteria, (ii) *Arthrobacter* CL28, (iii) *Variovorax* CL14 and (iv) *Arthrobacter* CL28 + *Variovorax* CL14. Each treatment was repeated in three separate agar plates with five tomato seedlings per plate. The experiment was repeated in two independent replicates.

#### Bacterial culture and plant inoculation.

All strains were grown in separate tubes, then washed and OD_600_ was adjusted to 0.01 before mixing and spreading (‘Bacterial culture and plant inoculation’ in ‘Tripartite plant–microorganism–microorganism experiments’). Four hundred μl of each bacterial treatment was spread on 20 × 20 agar plates containing JM agar with no sucrose.

#### In vitro plant growth conditions.

We used tomato cultivar Heinz 1706 seeds. All seeds were soaked in sterile distilled water for 15 min, then surface-sterilized with 70% bleach, 0.2% Tween-20 for 15 min, and rinsed 5 times with sterile distilled water to eliminate any seed-borne microorganisms on the seed surface. Seeds were stratified at 4 °C in the dark for 2 days. Plants were germinated on vertical square 10 × 10 cm agar plates with JM containing 0.5% sucrose, for 7 days. Then, 5 plants were transferred to each of the synthetic-community-inoculated agar plates. Upon collection, root morphology was measured (‘Root and shoot image analysis’ in ‘Deconstructing the synthetic community to four modules of co-occurring strains’).

#### Primary root elongation analysis.

Differences between treatments were indicated using the confidence letter display derived from the Tukey’s post hoc test from an ANOVA model.

### Determination of *Arthrobacter* CL28 colony forming units from roots

This section relates to Extended Data Fig. 6.

*Arabidopsis* seedlings were inoculated with (i) *Arthrobacter* CL28 alone, (ii) *Arthrobacter* CL28 + *Variovorax* CL14 or (iii) *Arthrobacter* CL28 + *Variovorax* B4, as described in ‘Bacterial culture and plant inoculation’ in ‘Tripartite plant–microorganism–microorganism experiments’. Each bacterial treatment included four separate plates, with nine seedlings in each plate. Upon collection, all seedlings were placed in pre-weighed 2-ml Eppendorf tubes containing 3 glass beads, 3 seedlings per tube (producing 12 data points per treatment). Roots were weighed, and then homogenized using a bead beater (MP Biomedicals). The resulting suspension was serially diluted, then plated on LB agar plates containing 50 μg/ml of apramycin to select for *Arthrobacter* CL28 colonies and colonies were counting after incubation of 48 h at 28 °C.

### *Arabidopsis* RNA-seq analysis

This section relates to Extended Data Fig. 7.

#### Detection of RGI-induced genes.

To measure the transcriptional response of the plant to the different synthetic community combinations, we used the R package DESeq2 v.1.22.1^44^. The raw count genes matrixes for the drop-out and tripartite experiments were used independently to define differentially expressed genes (DEGs). For the analysis of both experiments we fitted the following model specification: abundance gene ~ synthetic community.

From the fitted models, we derived the following contrasts to obtain DEGs. A gene was considered differentially expressed if it had a *q*-value < 0.1. For the tripartite system (‘Tripartite plant–microorganism–microorganism experiments’), we performed the following contrasts: *Arthrobacter* CL28 versus no bacteria (NB) and *Arthrobacter* CL28 versus *Arthrobacter* CL28 co-inoculated with *Variovorax* CL14. The logic behind these two contrasts was to identify genes that were induced in RGI plants (*Arthrobacter* CL28 versus NB) and repressed by *Variovorax* CL14. For the drop-out system (‘*Variovorax* drop-out experiment’), we performed the following contrasts, *Variovorax* drop-out versus NB, and *Variovorax* drop-out versus full synthetic community. The logic behind these two contrasts was identical to the tripartite system: to identify genes that are associated with the RGI phenotype (*Variovorax* drop-out versus NB contrast) and repressed when *Variovorax* are present (*Variovorax* drop-out versus full synthetic community contrast).

For visualization purposes, we applied a variance stabilizing transformation (DESeq2) to the raw count gene matrix. We then standardized each gene expression (*z*-score) along the samples. We subset DEGs from this standardized matrix and calculated the mean *z*-score expression value for each synthetic community treatment.

To identify the tissue-specific expression profile of the 18 intersecting genes between the tripartite and drop-out systems, we downloaded the spatial expression profile of each gene from the Klepikova atlas^25^ using the bio-analytic resource of plant biology platform. Then, we constructed a spatial expression matrix of the 18 genes and computed pairwise Pearson correlation between all pairs of genes. Finally, we applied hierarchical clustering to this correlation matrix.

#### Comparison with acute auxin response dataset.

This section relates to Extended Data Fig. 7d. We applied the variance stabilizing transformation (DESeq2) to the raw count gene matrix. We then standardized each gene expression (*z*-score) along the samples. From this matrix, we subset 12 genes that in a previous study^26^ exhibited the highest fold change between auxin-treated and untreated samples. Finally, we calculated the mean *z*-score expression value of each of these 12 genes across the synthetic community treatments. We estimated the statistical significance of the trend of these 12 genes between a pair of synthetic community treatments (full synthetic community versus *Variovorax* drop-out, *Arthrobacter* CL28 versus *Arthrobacter* CL28 plus *Variovorax* CL14) using a permutation approach: we estimated a *P* value by randomly selecting 12 genes 10,000 times from the expression matrix and comparing the mean expression between the two synthetic community treatments (for example, full synthetic community versus *Variovorax* drop-out) with the actual mean expression value from the 12 genes reported as robust auxin markers.

### Measuring the ability of *Variovorax* to attenuate RGI induced by small molecules IAA, 2,4-dichlorophenoxyacetic acid, ethylene (ACC), cytokinins (zeatin and 6-benzylaminopurine) and flagellin 22 peptide (flg22)

This section relates to Fig. 3a, Extended Data Fig. 8a.

#### Bacterial culture and plant inoculation.

We embedded each of the following compounds in JM plates: 100 nM IAA (Sigma), 100 nM 1- ACC) (Sigma), 100 nM 2,4-dichlorophenoxyacetic acid (Sigma), 100 nM flg22 (PhytoTech labs), 100 nM 6-benzylaminopurine (BAP) (Sigma) and 100 nM zeatin (Sigma). As some of these compound stocks were initially solubilized in ethanol, we included comparable amounts of ethanol in the control treatments. Plates with each compound were inoculated with one of the *Variovorax* strains CL14, MF160, B4 or YR216 or with *Burkholderia* CL11. These strains were grown in separate tubes, then washed and OD_600_ was adjusted to 0.01 before spreading 100 μl on plates. In addition, we included uninoculated controls for each compound. We also included unamended JM plates inoculated with the RGI-inducing *Arthrobacter* CL28 co-inoculated with each of the *Variovorax* or *Burkholderia* strains, or alone. Thus, the experiment included 42 individual treatments. The experiment was repeated twice, with three independent replicates per experiment. Upon collection, root morphology was measured (‘Root and shoot image analysis’ in ‘Deconstructing the synthetic community to four modules of co-occurring strains’).

#### Primary root elongation analysis.

Primary root elongation was compared between bacterial treatments within each of RGI treatments tested. Differences between treatments were estimated as described in ‘Primary root elongation analysis’ in ‘Tripartite plant–microorganism–microorganism experiments’. We plotted the estimated means with 95% confidence interval of each bacterial treatment across the different RGI treatments.

### In vitro growth of *Variovorax*

This section relates to Fig. 4b, Extended Data Figs. 8b, 9d, e.

*Variovorax* CL14 was grown in 5-ml cultures for 40 h at 28 °C in 1 × M9 minimal salts medium (Sigma M6030) supplemented with 2 mM MgSO_4_, 0.1 mM CaCl_2_, 10 μM FeSO_4_, and a carbon source: either 15 mM succinate alone, 0.4 mM IAA with 0.5% ethanol for IAA solubilization, or both. Optical density at 600 nm and IAA concentrations were measured at six time points. IAA concentrations were measured using the Salkowski method modified from ref.^56^. One hundred μl of Salkowski reagent (10 mM FeCl_3_ in 35% perchloric acid) was mixed with 50 μl culture supernatant or IAA standards and colour was allowed to develop for 30 min before measuring the absorbance at 530 nm.

### Measuring plant auxin response in vivo using a bioreporter line

This section relates to Extended Data Fig. 8c, d.

#### Bacterial culture and plant-inoculation.

Seven-day-old transgenic *Arabidopsis* seedlings expressing the *DR5::GFP* reporter construct^57^ were transferred onto plates containing: (i) 100 nM IAA, (ii) *Arthrobacter* CL28, (iii) 100 nM IAA + *Variovorax* CL14, (iv) *Arthrobacter* CL28 + *Variovorax* CL14, (v) uninoculated plates. For treatments (ii) and (iii), Bacterial strains were grown in separate tubes, then washed and OD_600_ was adjusted to 0.01. For treatment (iv), OD-adjusted cultures were mixed in 1:1 ratios and spread onto agar before seedling transfer.

#### Fluorescence microscopy.

GFP fluorescence in the root elongation zone of 8–10 plants per treatment were visualized using a Nikon Eclipse 80i fluorescence microscope at days 1, 3, 6, 9 and 13 after inoculation. The experiment was performed in two independent replicates.

From each root imaged, 10 random 30 × 30 pixel squares were sampled and average GFP intensity was measured using imageJ^45^. Treatments were compared within each time point using ANOVA tests with Tukey’s post hoc in the R base package emmeans. For visualization purposes, we plotted the estimated means of each bacterial across the different time points.

### Measuring the dual role of auxin and ethylene perception in synthetic-community-induced RGI

This section relates to Fig. 3b.

#### Bacterial culture and plant inoculation.

We transferred four 7-day-old wild-type seedling and four *axr2-1*seedlings to each plate in this experiment. The plates contained one of five bacterial treatments: (i) *Arthrobacter* CL28, (ii) *Arthrobacter* CL28 + *Variovorax* CL14, (iii) *Variovorax* drop-out synthetic community, (iv) full synthetic community, (v) uninoculated, prepared as described in ‘Bacterial culture and plant inoculation’ in ‘*Variovorax* drop-out under varying abiotic contexts’, and in ‘Bacterial culture and plant inoculation’ in ‘Measuring plant auxin response in vivo using a bioreporter line’. Plates were placed vertically inside sealed 86 × 68 cm Ziploc bags. In one of the bags, we placed an open water container with 80 2.5 g sachets containing 0.014% 1-MCP (Ethylene Buster, Chrystal International BV). In the second bag we added, as a control, an open water container. Both bags were placed in the growth chamber for 12 days. After 6 days of growth, we added 32 additional sachets to the 1-MCP-treated bag to maintain 1-MCP concentrations in the air. Upon collection, root morphology was measured (‘Root and shoot image analysis’ in ‘Deconstructing the synthetic community to four modules of co-occurring strains’).

#### Primary root elongation analysis.

Primary root elongation was standardized to the no-bacteria control of each genotype, and compared between genotype and 1-MCP treatments within the *Arthrobacter* CL28 treatment and within the *Variovorax* drop-out synthetic community treatment, independently. Differences between treatments were estimated as described in ‘Primary root elongation analysis’ in ‘Tripartite plant–microorganism–microorganism experiments’. We plotted the estimated means with 95% confidence interval of each bacterial treatment across the four genotypes. We calculated the IQR for the full and *Arthrobacter* CL28 with *Variovorax* CL14 treatments, pooling the four genotypes and MCP treatments.

### Preparation of binarized plant images

This section relates to Figs. 1c, 2b, Extended Data Fig. 2c.

To present representative root images, we increased contrast and subtracted background in ImageJ, then cropped the image to select representative roots. Neighbouring roots were manually erased from the cropped image.

### Mining *Variovorax* genomes for auxin degradation operons and ACC-deaminase genes and comparative genomics of *Variovorax* genomes against the other synthetic community members

This section relates to Extended Data Fig. 5a, Supplementary Table 4.

We used local alignment (BLASTp)^58^ to search for the presence of the 10 genes (*iacA*, *iacB*, *iacC*, *iacD*, *iacE*, *iacF*, *iacG*, *iacH*, *iacI* and *iacY*) from a previously characterized auxin degradation operon in a different genus^17^ across the 10 *Variovorax* isolates in our synthetic community. A minimal set of seven of these genes (*iacA*, *iacB*, *iacC*, *iacD*, *iacE*, *iacF* and *iacI*) was shown to be necessary and sufficient for auxin degradation^17^. We identified homologues for these genes across the *Variovorax* phylogeny (Extended Data Fig. 5a) at relatively low sequence identity (27–48%). Two genes of the minimal set of seven genes did not have any homologues in most *Variovorax* genomes (*iacB* and *iacI*). In addition to the *iac* operon, we scanned the genomes for the auxin degradation operon described in ref.^59^ and could not identify it in any of the *Variovorax* isolates.

We also searched for the ACC deaminase gene by looking for the KEGG orthology identifier K01505 (1-aminocyclopropane-1-carboxylate deaminase) across the IMG annotations available for all our genomes.

We used orthofinder v.2.2.1^60^ to construct orthogroups (group of homologue sequences) using all the coding sequences of the 10 *Variovorax* isolates included in the full 185-member synthetic community. We aligned each orthogroup using MAFFT v.707^53^ and proceeded to build hidden Markov model (HMM) profiles from the alignments using hmmbuild v.3.1b2^52^. We then used the HMM profiles that consist of core genes (present in the 10 isolates) in the *Variovorax* genus and scanned the 175 remaining genomes in the synthetic community for these HMM profiles using hmmsearch v.31.b2^52^. We then selected orthogroups that were less than 5% prevalent in the 175 remaining isolates scanned. Finally, taking the *Variovorax* CL14 genome as a reference, we built hotspots of physically adjacent selected orthogroups. We used an iterative approach that extended adjacent orthogroups if they were less than 10 kb from each other. As a final filtering step, we selected hotspots that contained more than 10 selected orthogroups.

### *Variovorax* CL14 RNA-seq in monoculture and in coculture with *Arthrobacter* CL28

This section relates to Fig. 4a, Extended Data Fig. 9b, c.

#### Bacterial culture.

*Variovorax* CL14 was grown either alone or in coculture with *Arthrobacter* CL28 in 5 ml of 1/10 2× YT medium (1.6 g/l tryptone, 1 g/l yeast extract, 0.5 g/l NaCl) in triplicate. The monoculture was inoculated at OD_600_ of 0.02 and the coculture was inoculated with OD_600_ of 0.01 of each strain. Cultures were grown at 28 °C to early stationary phase (approximately 22 h) and cells were collected by centrifugation at 4,100*g* for 15 min and frozen at −80 °C before RNA extraction.

#### RNA extraction and RNA-seq.

Cells were lysed for RNA extraction using TRIzol Reagent (Invitrogen) according to the manufacturer instructions. Following cell lysis and phase separation, RNA was purified using the RNeasy Mini kit (Qiagen) including the optional on column DNase Digestion with the RNase-Free DNase Set (Qiagen). Total RNA quality was confirmed on the 2100 Bioanalyzer instrument (Agilent) and quantified using a Qubit 2.0 fluorometer (Invitrogen). RNA-seq libraries were prepared using the Universal Prokaryotic RNA-Seq, Prokaryotic AnyDeplete kit (Tecan, formerly NuGEN). Libraries were pooled and sequenced on the Illumina HiSeq4000 platform to generate 50-bp single-end reads.

#### RNA-seq analysis.

We mapped the generated raw reads to the *Variovorax* CL14 genome (fasta file available on https://github.com/isaisg/variovoraxRGI/blob/master/rawdata/2643221508.fna) using bowtie2^61^ with the ‘very sensitive’ flag. We then counted hits to each individual coding sequence annotated for the *Variovorax* CL14 genome using the function featureCounts from the R package Rsubread, inputting the *Variovorax* CL14 gff file (available on https://github.com/isaisg/variovoraxRGI/blob/master/rawdata/2643221508.gff) and using the default parameters with the flag allowMultiOverlap = FALSE. Finally, we used DESeq2 to estimate DEGs between treatments with the corresponding fold change estimates and FDR-adjusted *P* values.

### *Variovorax* CL14 genomic library construction and screening

This section relates to Fig. 4a, Extended Data Fig. 9b, Supplementary Table 5.

#### Library construction.

High-molecular -eight *Variovorax* CL14 genomic DNA was isolated by phenol–chloroform extraction. This genomic DNA was partially digested with Sau3A1 (New England Biolabs), and separated on the BluePippin (Sage Science) to isolate DNA fragments >12.5 kb. Vector backbone was prepared by amplifying pBBR-1MCS2^62^ using Phusion polymerase (New England Biolabs) with primers JMC277–JMC278 (Supplementary Table 6), digesting the PCR product with BamHI-HF (New England Biolabs), dephosphorylating with Quick CIP (New England Biolabs), and gel extracting using the QIAquick Gel Extraction Kit (Qiagen). The prepared *Variovorax* CL14 genomic DNA fragments were ligated to the prepared pBBR1-MCS2 vector backbone using ElectroLigase (New England Biolabs) and transformed by electroporation into NEB 10-beta Electrocompetent *E. coli* (New England Biolabs). Clones were selected by blue–white screening on LB plates containing 1.5% agar, 50 μg/ml kanamycin, 40 μg/ml X-gal (5-bromo-4-chloro-3-indolyl-β-d-galactopyranoside), and 1 mM isopropyl β-d-1-thiogalactopyranoside (IPTG) at 37 °C. White colonies were screened by colony PCR using Taq polymerase and JMC247–JMC270 primers (Supplementary Table 6) to eliminate clones with small inserts. The screened library clones were picked into LB medium + 50 μg/ml kanamycin, grown at 37 °C, and stored at −80 °C in 20% glycerol. The *Variovorax* CL14 genomic library comprises approximately 3,500 clones with inserts >12.5 kb in vector pBBR1-MCS2 in NEB 10-beta *E. coli*.

#### Library screening for IAA degradation.

To screen the *Variovorax* CL14 genomic library for IAA degradation, the *E. coli* clones were grown in LB medium containing 50 μg/ml kanamycin, 1 mM IPTG, 0.05 mg/ml IAA, and 0.25% ethanol from IAA solubilization for 3 days at 37 °C. Salkowski reagent (10 mM FeCl_3_ in 35% perchloric acid) was mixed with culture supernatant 2:1 and colour was allowed to develop for 30 min before measuring the absorbance at 530 nm. Two clones from the library (plate 8A well E8 and plate2A well F10, henceforth vector 1 and vector 2, respectively) were identified as degrading IAA. The *Variovorax* CL14 genes contained in vectors 1 and 2 were inferred by isolating plasmid from these clones using the ZymoPURE II Plasmid Midiprep Kit (Zymo Research) and Sanger-sequencing the insert ends using primers JMC247 and JMC270 (Supplementary Table 6). Double digest of the purified plasmids with SacI and EcoRV confirmed the size of the inserts. Vector 1 contains a 35-kb insert and vector 2 contains a 15-kb insert (nucleotide coordinates 29,100–64,406 and 52,627–67,679, respectively, from *Variovorax* CL14 scaffold Ga0102008_10005) (Fig. 4a, Extended Data Fig. 9b). The genes in both inserts are in the same direction as the IPTG0inducible *Lac* promoter used to drive *LacZ*-alpha expression for blue–white screening on pBBR1-MCS2.

### *Acidovorax* Root219::EV and *Acidovorax* root219::V2 construction and screening

This section relates to Fig. 4b–d, Extended Data Fig. 9b.

Triparental mating was used to mobilize vector 2 or control EV pBBR1-MCS2 from *E. coli* to *Acidovorax* root219. Donor NEB 10-beta *E. coli* containing the vector for conjugation and helper strain *E. coli* pRK2013^63^ were grown in LB medium containing 50 μg/ml kanamycin at 37 °C. *Acidovorax* root219 was grown in 2× YT medium containing 100 μg/ml ampicillin at 28 °C. Bacteria were washed 3 times with 2× YT medium without antibiotics, mixed in a ratio of approximately 1:1:10 donor:helper:recipient, centrifuged and resuspended in 1/10 the volume and plated as a pool on LB agar plates without antibiotics and grown at 28 °C. Eighteen to thirty h later, exconjugantes were streaked on LB agar plates containing 50 μg/ml kanamycin and 100 μg/ml ampicillin to select only *Acidovorax* root219 containing the conjugated vector. The resulting strains are designated *Acidovorax* root219::EV containing empty vector pBBR1-MCS2 and *Acidovorax* root219::V2 containing vector 2. In vitro IAA degradation was performed as in ‘In vitro growth of *Variovorax*’ using M9 medium with carbon sources: 15 mM succinate, 0.1 mg/ml IAA and 0.5% ethanol with the addition of 50 μg/ml kanamycin and 1 mM IPTG. Primary root elongation measurement was performed as described in ‘Root and shoot image analysis’ in ‘Deconstructing the synthetic community to four modules of co-occurring strains’, on MS medium with 1 mM IPTG and RGI induced by either 100 nM IAA or *Arthrobacter* CL28. *Acidovorax* root219::V2 root colonization was compared to *Variovorax* CL14 colonization by plating a subset of ground root samples from the root elongation experiment (see ‘Determination of *Arthrobacter* CL28 colony forming units from roots’ for root collection and processing protocol) on LB agar plates containing 100 μg/ml ampicillin, for which *Arthrobacter* CL28 is susceptible and *Variovorax* CL14 and *Acidovorax* Root219 are not. Number of colony-forming units (CFUs) was normalized to root weight (Extended Data Fig. 9d).

### *Variovorax* hotspot 33 knockout construction and screening

This section relates to Fig. 4b–d, Extended Data Fig. 9b.

The unmarked deletion mutant *Variovorax* CL14 Δ2643613653–2643613677 (*Variovorax* CL14 ΔHS33) was constructed based on a genetic system developed for *Burkholderia* spp. and its suicide vector pMo130^64^

#### Knockout suicide vector pJMC158 construction.

The vector backbone was amplified from pMo130 using primers JMC203–JMC204 (Supplementary Table 6) with Q5 DNA polymerase (New England Biolabs), cleaned up and treated with DpnI (New England Biolabs). One-kb regions for homologous recombination flanking *Variovorax* CL14 genes 2643613653–2643613677 were amplified using Q5 Polymerase (New England Biolabs) and primers JMC533–JMC534 and JMC535–JMC536 (Supplementary Table 6). The vector was assembled with Gibson Assembly Mastermix (New England Biolabs) at 50 °C for 1 h, transformed into NEB 5-alpha chemically competent *E. coli* (New England Biolabs), and plated on LB agar with 50 μg/ml kanamycin. pJMC158 DNA was isolated from a clone using the ZR Plasmid Miniprep Classic Kit (Zymo Research), sequence confirmed, and transformed into biparental mating strain *E. coli* WM3064. *E. coli* strain WM3064 containing pJMC158 was maintained on LB containing 50 μg/ml kanamycin and 0.3 mM diaminopimelic acid (DAP) at 37 °C.

#### Conjugative transfer of pJMC158 into Variovorax CL14.

For biparental mating, *E. coli* WM3064 containing pJMC158 was grown as above, and *Variovorax* CL14 was grown in 2 × YT medium containing 100 μg/ml ampicillin. Each strain was washed separately 3 times with 2 × YT medium, then mixed at ratios between 1:1–1:10 donor:recipient, centrifuged and resuspended in approximately 1/10 the volume and plated in a single pool on LB agar containing 0.3 mM DAP and grown at 28 °C overnight. Exconjugants were streaked onto LB plates containing 100 μg/ml ampicillin, 50 μg/ml kanamycin lacking DAP and grown at 28 °C to select *Variovorax* CL14 strains that incorporated suicide vector pJMC158. First crossover strains were subsequently purified once by restreaking and then individual colonies grown in LB with 100 μg/ml ampicillin, 50 μg/ml kanamycin.

#### Resolution of pJMC158 integration and knockout strain purification and verification.

To resolve the integration of pJMC158, first crossover strains were grown once in LB medium containing 100 μg/ml ampicillin and 1 mM IPTG then plated on medium containing 10 g/l tryptone, 5 g/l yeast extract, 100 g/l sucrose, 1.5% agar, 100 μg/ml ampicillin and 1 mM IPTG. Colonies were picked into the same liquid medium and grown once. The resulting strains were screened by PCR using Q5 polymerase for deletion of genes 2643613653–2643613677 using primers JMC568–JMC569 (Supplementary Table 6). These strains were subsequently plate-purified at least 3 times on LB 100 μg/ml ampicillin plates. To ensure strain purity, PCR primers were designed to amplify from outside into the genes that were deleted (primer pairs JMC571–JMC569 and JMC568–JMC570) (Supplementary Table 6). These PCR reactions were performed using Q5 polymerase with wild-type *Variovorax* CL14 as a control. All genomic DNA used for screening PCR was isolated using the Quick-DNA miniprep kit (Zymo Research). The resulting knockout strain was designated *Variovorax* CL14 ΔHS33.

#### Screening of *Variovorax* CL14ΔHS33.

In vitro IAA degradation was performed as in ‘In vitro growth of *Variovorax*’ using M9 medium with carbon sources: 15 mM succinate, 0.1 mg/ml IAA and 0.5% ethanol. Primary root elongation measurement was performed as described in ‘Root and shoot image analysis’ in ‘Deconstructing the synthetic community to four modules of co-occurring strains’, on MS medium with 1mM IPTG and RGI induced by either 100 nM IAA or *Arthrobacter* CL28. To control for pleiotropic colonization effects, CFU counts were obtained for both CL14 and CL14 ΔHS33 in binary-association with the plant (see ‘Determination of *Arthrobacter* CL28 colony-forming units from roots’ for root collection and processing protocol). CL14 ΔHS33 colonization was not impaired (Extended Data Fig. 9e).

### Screening existing 16S rRNA census data for *Variovorax*

This section relates to Extended Data Fig. 10.

#### Natural *Arabidopsis* populations across Europe^6^.

We used the DADA2^65^ pipeline to create amplicon sequence variants (ASVs) from the raw reads published in ref.^6^. We then used the naive Bayes classifier implemented in mothur^66^ to taxonomically classify each ASV using the SILVA 132 database^67^. To determine prevalence and relative abundance of ASVs, We followed the same approach as in ref.^6^. The bacterial ASV table was restricted to samples having >1,000 reads. The table was transformed to relative abundance (RA) by dividing each value in a sample by the total reads in that sample. An ASV was considered to be present in a sample if it had an RA >0.01% in that particular sample. To calculate the average relative abundance (*y*-axis in Extended Data Figs. 10a, b) independently of prevalence, we calculated the mean RA using only the sample for which each ASV was considered present. We used the same classification scheme to colour the ASVs across the scatter plots shown. ASVs present in >80% of the sites on average are considered as widespread ASVs.

#### Different plant species in the same soil^4^.

We used the ASV table provided within ref.^4^ and followed the same pipeline described in ‘Natural *Arabidopsis* populations across Europe’. For each sample in the dataset, an ASV was considered to be present if that ASV had an RA >0.01% in that particular sample. To calculate the average relative abundance (*y*-axis in Extended Data Figs. 10a, b) independently of prevalence, we calculated the mean RA using only the samples for which each ASV was considered present. For each one of the thirty plant species in this data set, an ASV was considered present in that plant species if it was present in >70% of samples coming from that plant species. An ASV was considered core if it was present in all 30 plant species surveyed in this experiment.

## Extended Data

## Supplementary Material

Supplementary Tables

## Data Availability

The 16S rRNA amplicon sequencing data associated with this study have been deposited in the NCBI Sequence Read Archive under the project accession PRJNA543313. The raw transcriptomic data have been deposited in the Gene Expression Omnibus under the accession GSE131158. We deposited all scripts and additional data structures required to reproduce the results of this study in the following GitHub repository: https://github.com/isaisg/variovoraxRGI. Source data are provided with this paper.

## References

[1] Castrillo G Root microbiota drive direct integration of phosphate stress and immunity. Nature 543, 513–518 (2017). 28297714 10.1038/nature21417 PMC5364063

[2] Durán P Microbial interkingdom interactions in roots promote *Arabidopsis* survival. Cell 175, 973–983.e14 (2018). 30388454 10.1016/j.cell.2018.10.020 PMC6218654

[3] Herrera Paredes S Design of synthetic bacterial communities for predictable plant phenotypes. PLoS Biol. 16, e2003962 (2018). 29462153 10.1371/journal.pbio.2003962 PMC5819758

[4] Fitzpatrick CR Assembly and ecological function of the root microbiome across angiosperm plant species. Proc. Natl Acad. Sci. USA 115, E1157–E1165 (2018). 29358405 10.1073/pnas.1717617115 PMC5819437

[5] Finkel OM The effects of soil phosphorus content on plant microbiota are driven by the plant phosphate starvation response. PLoS Biol. 17, e3000534 (2019). 31721759 10.1371/journal.pbio.3000534 PMC6876890

[6] Thiergart T Root microbiota assembly and adaptive differentiation among European *Arabidopsis* populations. Nat. Ecol. Evol 4, 122–131 (2020). 31900452 10.1038/s41559-019-1063-3

[7] Hogenhout SA, Van der Hoorn RAL, Terauchi R & Kamoun S Emerging concepts in effector biology of plant-associated organisms. Mol. Plant Microbe Interact 22, 115–122 (2009). 19132864 10.1094/MPMI-22-2-0115

[8] Mylona P, Pawlowski K & Bisseling T Symbiotic nitrogen fixation. Plant Cell 7, 869–885 (1995). 12242391 10.1105/tpc.7.7.869 PMC160880

[9] Ludwig-Müller J Bacteria and fungi controlling plant growth by manipulating auxin: balance between development and defense. J. Plant Physiol 172, 4–12 (2015). 25456606 10.1016/j.jplph.2014.01.002

[10] Carlström CI Synthetic microbiota reveal priority effects and keystone strains in the *Arabidopsis* phyllosphere. Nat. Ecol. Evol 3, 1445–1454 (2019). 31558832 10.1038/s41559-019-0994-z PMC6774761

[11] Faure D, Vereecke D & Leveau JHJ Molecular communication in the rhizosphere. Plant Soil 321, 279–303 (2009).

[12] Leadbetter JR & Greenberg EP Metabolism of acyl-homoserine lactone quorum-sensing signals by *Variovorax paradoxus*. J. Bacteriol 182, 6921–6926 (2000). 11092851 10.1128/jb.182.24.6921-6926.2000 PMC94816

[13] Leveau JHJ & Lindow SE Utilization of the plant hormone indole-3-acetic acid for growth by *Pseudomonas putida* strain 1290. Appl. Environ. Microbiol 71, 2365–2371 (2005). 15870323 10.1128/AEM.71.5.2365-2371.2005 PMC1087548

[14] Zúñiga A Quorum sensing and indole-3-acetic acid degradation play a role in colonization and plant growth promotion of *Arabidopsis thaliana* by *Burkholderia phytofirmans* PsJN. Mol. Plant Microbe Interact 26, 546–553 (2013). 23301615 10.1094/MPMI-10-12-0241-R

[15] Sun S-L The plant growth-promoting rhizobacterium *Variovorax boronicumulans* CGMCC 4969 regulates the level of indole-3-acetic acid synthesized from indole-3-acetonitrile. Appl. Environ. Microbiol 84, e00298–18 (2018). 29884755 10.1128/AEM.00298-18 PMC6070764

[16] Gilbert S Bacterial production of indole related compounds reveals their role in association between duckweeds and endophytes. Front. Chem 6, 265 (2018). 30050896 10.3389/fchem.2018.00265 PMC6052042

[17] Donoso R Biochemical and genetic bases of indole-3-acetic acid (auxin phytohormone) degradation by the plant-growth-promoting rhizobacterium *Paraburkholderia phytofirmans* PsJN. Appl. Environ. Microbiol 83, e01991–16 (2016). 27795307 10.1128/AEM.01991-16 PMC5165117

[18] Leveau JHJ & Gerards S Discovery of a bacterial gene cluster for catabolism of the plant hormone indole 3-acetic acid. FEMS Microbiol. Ecol 65, 238–250 (2008). 18205812 10.1111/j.1574-6941.2008.00436.x

[19] Brumos J Local auxin biosynthesis is a key regulator of plant development. Dev. Cell 47, 306–318.e5 (2018). 30415657 10.1016/j.devcel.2018.09.022

[20] Levy A Genomic features of bacterial adaptation to plants. Nat. Genet 50, 138–150 (2018). 10.1038/s41588-017-0012-9 PMC5957079 29255260

[21] Kremer JM FlowPot axenic plant growth system for microbiota research. Preprint at https://www.biorxiv.org/content/10.1101/254953v1 (2018).

[22] Clauss MJ & Aarssen LW Phenotypic plasticity of size–fecundity relationships in *Arabidopsis thaliana*. J. Ecol 82, 447 (1994).

[23] Abreu ME & Munné-Bosch S Salicylic acid deficiency in *NahG* transgenic lines and *sid2* mutants increases seed yield in the annual plant *Arabidopsis* thaliana. J. Exp. Bot 60, 1261–1271 (2009). 19188277 10.1093/jxb/ern363 PMC2657544

[24] Bai Y Functional overlap of the *Arabidopsis* leaf and root microbiota. Nature 528, 364–369 (2015). 26633631 10.1038/nature16192

[25] Klepikova AV, Kasianov AS, Gerasimov ES, Logacheva MD & Penin AA A high resolution map of the *Arabidopsis thaliana* developmental transcriptome based on RNA-seq profiling. Plant J. 88, 1058–1070 (2016). 27549386 10.1111/tpj.13312

[26] Uchida N Chemical hijacking of auxin signaling with an engineered auxin–TIR1 pair. Nat. Chem. Biol 14, 299–305 (2018). 29355850 10.1038/nchembio.2555 PMC5812785

[27] Takase T *ydk1-D*, an auxin-responsive *GH3* mutant that is involved in hypocotyl and root elongation. Plant J. 37, 471–483 (2004). 14756757 10.1046/j.1365-313x.2003.01973.x

[28] Chen L, Dodd IC, Theobald JC, Belimov AA & Davies WJ The rhizobacterium *Variovorax paradoxus* 5C-2, containing ACC deaminase, promotes growth and development of *Arabidopsis thaliana* via an ethylene-dependent pathway. J. Exp. Bot 64, 1565–1573 (2013). 23404897 10.1093/jxb/ert031 PMC3617834

[29] Cary AJ, Liu W & Howell SH Cytokinin action is coupled to ethylene in its effects on the inhibition of root and hypocotyl elongation in *Arabidopsis thaliana* seedlings. Plant Physiol. 107, 1075–1082 (1995). 7770519 10.1104/pp.107.4.1075 PMC157239

[30] Gómez-Gómez L, Felix G & Boller T A single locus determines sensitivity to bacterial flagellin in *Arabidopsis thaliana*. Plant J. 18, 277–284 (1999). 10377993 10.1046/j.1365-313x.1999.00451.x

[31] Robles L, Stepanova A & Alonso J Molecular mechanisms of ethylene–auxin interaction. Mol. Plant 6, 1734–1737 (2013). 23935009 10.1093/mp/sst113

[32] Nagpal P *AXR2* encodes a member of the Aux/IAA protein family. Plant Physiol. 123, 563–574 (2000). 10859186 10.1104/pp.123.2.563 PMC59024

[33] Hall AE, Findell JL, Schaller GE, Sisler EC & Bleecker AB Ethylene perception by the ERS1 protein in *Arabidopsis*. Plant Physiol. 123, 1449–1458 (2000). 10938361 10.1104/pp.123.4.1449 PMC59101

[34] Gould SJ & Vrba ES Exaptation—a missing term in the science of form. Paleobiology 8, 4–15 (1982).

[35] Bardoel BW Pseudomonas evades immune recognition of flagellin in both mammals and plants. PLoS Pathog. 7, e1002206 (2011). 21901099 10.1371/journal.ppat.1002206 PMC3161968

[36] Lundberg DS, Yourstone S, Mieczkowski P, Jones CD & Dangl JL Practical innovations for high-throughput amplicon sequencing. Nat. Methods 10, 999–1002 (2013). 23995388 10.1038/nmeth.2634

[37] Yourstone SM, Lundberg DS, Dangl JL & Jones CD MT-Toolbox: improved amplicon sequencing using molecule tags. BMC Bioinformatics 15, 284 (2014). 25149069 10.1186/1471-2105-15-284 PMC4153912

[38] Joshi N & Fass J Sickle: a sliding-window, adaptive, quality-based trimming tool for FastQ files (version 1.33), https://github.com/najoshi/sickle (2011).

[39] Edgar RC Search and clustering orders of magnitude faster than BLAST. Bioinformatics 26, 2460–2461 (2010). 20709691 10.1093/bioinformatics/btq461

[40] Edgar RC UPARSE: highly accurate OTU sequences from microbial amplicon reads. Nat. Methods 10, 996–998 (2013). 23955772 10.1038/nmeth.2604

[41] Wang Q, Garrity GM, Tiedje JM & Cole JR Naive Bayesian classifier for rapid assignment of rRNA sequences into the new bacterial taxonomy. Appl. Environ. Microbiol 73, 5261–5267 (2007). 17586664 10.1128/AEM.00062-07 PMC1950982

[42] DeSantis TZ Greengenes, a chimera-checked 16S rRNA gene database and workbench compatible with ARB. Appl. Environ. Microbiol 72, 5069–5072 (2006). 16820507 10.1128/AEM.03006-05 PMC1489311

[43] Oksanen J Vegan: Community Ecology Package, R package version 2.5–6, https://CRAN.R-project.org/package=vegan (2019).

[44] Love MI, Huber W & Anders S Moderated estimation of fold change and dispersion for RNA-seq data with DESeq2. Genome Biol. 15, 550 (2014). 25516281 10.1186/s13059-014-0550-8 PMC4302049

[45] Schindelin J Fiji: an open-source platform for biological-image analysis. Nat. Methods 9, 676–682 (2012). 22743772 10.1038/nmeth.2019 PMC3855844

[46] Lenth R emmeans: Estimated Marginal Means, aka Least-Squares Means, R package version 1.4.7, https://CRAN.R-project.org/package=emmeans (2020).

[47] Logemann J, Schell J & Willmitzer L Improved method for the isolation of RNA from plant tissues. Anal. Biochem 163, 16–20 (1987). 2441623 10.1016/0003-2697(87)90086-8

[48] Bolger AM, Lohse M & Usadel B Trimmomatic: a flexible trimmer for Illumina sequence data. Bioinformatics 30, 2114–2120 (2014). 24695404 10.1093/bioinformatics/btu170 PMC4103590

[49] Kim D, Langmead B & Salzberg SL HISAT: a fast spliced aligner with low memory requirements. Nat. Methods 12, 357–360 (2015). 25751142 10.1038/nmeth.3317 PMC4655817

[50] Liao Y, Smyth GK & Shi W The Subread aligner: fast, accurate and scalable read mapping by seed-and-vote. Nucleic Acids Res. 41, e108 (2013). 23558742 10.1093/nar/gkt214 PMC3664803

[51] Ewels P, Magnusson M, Lundin S & Käller M MultiQC: summarize analysis results for multiple tools and samples in a single report. Bioinformatics 32, 3047–3048 (2016). 27312411 10.1093/bioinformatics/btw354 PMC5039924

[52] Wheeler TJ & Eddy SR nhmmer: DNA homology search with profile HMMs. Bioinformatics 29, 2487–2489 (2013). 23842809 10.1093/bioinformatics/btt403 PMC3777106

[53] Katoh K & Standley DM MAFFT multiple sequence alignment software version 7: improvements in performance and usability. Mol. Biol. Evol 30, 772–780 (2013). 23329690 10.1093/molbev/mst010 PMC3603318

[54] Capella-Gutiérrez S, Silla-Martínez JM & Gabaldón T trimAl: a tool for automated alignment trimming in large-scale phylogenetic analyses. Bioinformatics 25, 1972–1973 (2009). 19505945 10.1093/bioinformatics/btp348 PMC2712344

[55] Price MN, Dehal PS & Arkin AP FastTree 2—approximately maximum-likelihood trees for large alignments. PLoS ONE 5, e9490 (2010). 20224823 10.1371/journal.pone.0009490 PMC2835736

[56] Gordon SA & Weber RP Colorimetric estimation of indoleacetic acid. Plant Physiol. 26, 192–195 (1951). 16654351 10.1104/pp.26.1.192 PMC437633

[57] Friml J Efflux-dependent auxin gradients establish the apical–basal axis of *Arabidopsis*. Nature 426, 147–153 (2003). 14614497 10.1038/nature02085

[58] Altschul SF, Gish W, Miller W, Myers EW & Lipman DJ Basic local alignment search tool. J. Mol. Biol 215, 403–410 (1990). 2231712 10.1016/S0022-2836(05)80360-2

[59] Ebenau-Jehle C Anaerobic metabolism of indoleacetate. J. Bacteriol 194, 2894–2903 (2012). 22447903 10.1128/JB.00250-12 PMC3370604

[60] Emms DM & Kelly S OrthoFinder: phylogenetic orthology inference for comparative genomics. Genome Biol. 20, 238 (2019). 31727128 10.1186/s13059-019-1832-y PMC6857279

[61] Langmead B & Salzberg SL Fast gapped-read alignment with Bowtie 2. Nat. Methods 9, 357–359 (2012). 22388286 10.1038/nmeth.1923 PMC3322381

[62] Kovach ME Four new derivatives of the broad-host-range cloning vector pBBR1MCS, carrying different antibiotic-resistance cassettes. Gene 166, 175–176 (1995). 8529885 10.1016/0378-1119(95)00584-1

[63] Figurski DH & Helinski DR Replication of an origin-containing derivative of plasmid RK2 dependent on a plasmid function provided in trans. Proc. Natl Acad. Sci. USA 76, 1648–1652 (1979). 377280 10.1073/pnas.76.4.1648 PMC383447

[64] Hamad MA, Zajdowicz SL, Holmes RK & Voskuil MI An allelic exchange system for compliant genetic manipulation of the select agents *Burkholderia pseudomallei* and *Burkholderia mallei*. Gene 430, 123–131 (2009). 19010402 10.1016/j.gene.2008.10.011 PMC2646673

[65] Callahan BJ DADA2: high-resolution sample inference from Illumina amplicon data. Nat. Methods 13, 581–583 (2016). 27214047 10.1038/nmeth.3869 PMC4927377

[66] Schloss PD Introducing mothur: open-source, platform-independent, community-supported software for describing and comparing microbial communities. Appl. Environ. Microbiol 75, 7537–7541 (2009). 19801464 10.1128/AEM.01541-09 PMC2786419

[67] Quast C The SILVA ribosomal RNA gene database project: improved data processing and web-based tools. Nucleic Acids Res. 41, D590–D596 (2013). 23193283 10.1093/nar/gks1219 PMC3531112

[68] Winter D An “Electronic Fluorescent Pictograph” browser for exploring and analyzing large-scale biological data sets. PLoS ONE 2, e718 (2007). 17684564 10.1371/journal.pone.0000718 PMC1934936

